# Phytoplankton Diversity in the Northern Adriatic Sea: Insights and Inconsistencies from Microscopy and Metabarcoding

**DOI:** 10.3390/biology15060487

**Published:** 2026-03-19

**Authors:** Giorgia Montali, Francesca Neri, Elisa Banchi, Federica Cerino, Timotej Turk Dermastia, Janja Francé, Patricija Mozetič, Angela Pelusi, Tiziana Romagnoli, Marika Ubaldi, Cecilia Totti, Stefano Accoroni

**Affiliations:** 1 Dipartimento di Scienze della Vita e dell’Ambiente, Università Politecnica delle Marche, Via Brecce Bianche, 60131 Ancona, Italy; s1112616@studenti.univpm.it (G.M.); t.romagnoli@univpm.it (T.R.); m.ubaldi@univpm.it (M.U.); c.totti@univpm.it (C.T.); 2 National Institute of Oceanography and Applied Geophysics—OGS, Via Piccard 54, 34151 Trieste, Italy; ebanchi@ogs.it (E.B.); fcerino@ogs.it (F.C.); apelusi@ogs.it (A.P.); 3 National Biodiversity Future Center, Piazza Marina 61, 90133 Palermo, Italy; 4 National Institute of Biology, Marine Biology Station Piran, Fornače 41, 6330 Piran, Slovenia; timotej.turkdermastia@nib.si (T.T.D.); janja.france@nib.si (J.F.); patricija.mozetic@nib.si (P.M.); 5 Dipartimento di Scienze della Terra e del Mare, Università degli Studi di Palermo, Via Archirafi 22, 90123 Palermo, Italy; 6 Fano Marine Center, The Inter-Institute Center for Research on Marine Biodiversity, Resources and Biotechnologies (FMC), Viale Adriatico 1/N, 61032 Fano, Italy; 7 Consorzio Interuniversitario per le Scienze del Mare, CoNISMa, 00196 Roma, Italy

**Keywords:** phytoplankton, diversity, environmental DNA, 18S, *rbcL*, Long-Term Ecological Research, amplicon sequencing

## Abstract

Phytoplankton is a key component of marine ecosystems and a sensitive indicator of environmental change. In this study, light microscopy (LM) and DNA metabarcoding (18S-V4, 18S-V9, and *rbcL*) were combined to assess differences in phytoplankton diversity and community structure across three LTER sites in the northern Adriatic Sea, and to evaluate the methodological effects on community assessment. A total of 329 genera and 527 species were recorded by integrating both the approaches. Metabarcoding (MB) revealed increased taxonomic richness than LM, particularly for dinoflagellates and small phytoflagellates, while LM was better for identifying the diatoms and coccolithophores. The *rbcL* marker improved the taxonomic resolution for the diatoms compared to the 18S regions. The proportion of species shared among the sites increased from 13% with LM to 33–42% with MB, suggesting that MB may effectively reduce the discrepancies observed when relying solely on LM. Cluster analysis performed on species-relative abundances grouped the samples by approaches rather than sites, showing that methodological variability exceeded the ecological differences. The relative abundance patterns differed between methods but became more comparable after applying correction factors based on the 18S rRNA gene copy numbers, particularly for the dinoflagellates. Overall, MB enhances biodiversity assessment and comparability among sites, while LM remains essential for morphological validation and for abundance assessment.

## Simple Summary

Phytoplankton plays a fundamental role in marine ecosystems and is widely used to assess environmental change. In this study, light microscopy and DNA metabarcoding approaches were used to investigate phytoplankton communities at three long-term monitoring sites in the northern Adriatic Sea. By combining the two approaches, more than 500 species were recorded. Metabarcoding detected substantially higher diversity, particularly among small and fragile organisms that are difficult to identify under a microscope, whereas microscopy was better for identifying larger species with distinctive morphology. Because species contain different amounts of genetic material, DNA data can overestimate or underestimate their true contribution to the community. Indeed, after applying correction factors that account for these differences, the DNA-based estimates became more consistent with the microscopy results for several phytoplankton groups. The differences among the samples were influenced more by the approach used than by the sampling location. Overall, combining these two approaches provides an accurate and robust picture of phytoplankton communities. In this way, it is possible to improve the reliability of marine biodiversity assessments and to support better environmental monitoring and management in coastal seas.

## 1. Introduction

Phytoplankton is a key component of marine ecosystems, representing the base of the oceanic food web and accounting for approximately 50% of global primary production [1,2]. Moreover, it plays an active role in major biogeochemical cycles, particularly the carbon cycle, by modulating CO_2_ exchange between oceans and the atmosphere [1,3,4]. Due to its high sensitivity to environmental changes, phytoplankton is considered an effective indicator of the ecological status of coastal ecosystems, which are increasingly exposed to anthropogenic pressures [5,6,7]. Indeed, the role of plankton as a key indicator of ecological status has been recognized by both the Water Framework Directive (WFD) and the Marine Strategy Framework Directive (MSFD).

The Northern Adriatic Sea (NAS) represents a particularly dynamic and ecologically significant region within the Mediterranean basin. Characterized by shallow waters, high riverine inputs, and marked seasonal variability, this area hosts highly productive coastal ecosystems that are subject to intense anthropogenic and climatic pressures [8,9]. These factors influence the spatial and temporal patterns of phytoplankton communities and offer valuable opportunities to investigate their responses to natural and anthropogenic drivers along environmental gradients.

Phytoplankton seasonal dynamics in the NAS have been dominated by diatom blooms in winter, spring, and autumn, with small phytoflagellates prevailing during the intervening periods [10,11,12,13]. Dinoflagellates become more abundant in spring and summer, while coccolithophores, though a minor component, persist throughout the year with a spring maximum [10,13].

In recent decades, this seasonal pattern has become increasingly affected by climate changes and extreme events, such as marine heatwaves, prolonged droughts and intense flooding, that are exerting a growing influence on phytoplankton communities [14,15].

Continuous monitoring of phytoplankton community structure and composition, as carried out at Long-Term Ecological Research (LTER) sites in the NAS (i.e., the Gulf of Venice, the Gulf of Trieste, the Po River delta, and the Senigallia-Susak Transect), is therefore crucial for detecting changes in marine ecosystems.

Traditionally, phytoplankton monitoring has relied on microscopy cell counts (LM), with the Utermöhl method [16] remaining the most widely used technique. Although LM analysis provides detailed information on the abundance and biomass of the main phytoplankton groups, it has several limitations in species-level identification, especially for cryptic or morphologically similar species, small or less abundant species, those in different life stages (e.g., cysts and auxospores) and fragile species that may be damaged by fixatives. Moreover, it requires time-consuming procedures, the need for advanced taxonomic expertise, and is highly affected by observer subjectivity [17,18,19].

To overcome these limitations, DNA metabarcoding (MB) has emerged over recent decades as a novel tool for biodiversity studies [20,21]. This molecular approach enables species identification, providing a faster, more standardized, and objective alternative to the traditional monitoring methods [20,21,22]. However, to ensure reliable results, MB requires the selection of appropriate genetic markers (barcodes) and primers capable of resolving taxonomic groups and species [23,24]. Moreover, the accuracy of the outcomes depends on the quality of reference databases [25,26] and the bioinformatic pipeline used [27,28].

The 18S rRNA gene is the most commonly used marker for eukaryotic identification [29,30,31,32,33,34,35]. However, a major limitation of this marker lies in the variability in gene copy number (GCN) among different organisms [21,36,37]. This is particularly relevant for groups such as dinoflagellates, which possess a high GCN, potentially leading to an overestimation of their relative abundance, and therefore to a bias in the assessment of community composition [38,39,40]. Indeed, a crucial limitation of MB for phytoplankton estimation is that it provides only relative abundances based on sequence counts, which do not directly reflect the actual number of cells in the water due to the compositional nature of high-throughput sequencing data and amplification bias [41].

As an attempt to improve the accuracy of relative abundance estimates derived from MB data, correction factors (CFs) have been proposed to account for the differences in 18S rRNA gene copy number across taxa (e.g., [42]). The effectiveness of CFs for protists is still limited by the incomplete availability of accurate 18S rRNA GCN data for many eukaryotic groups [40,43] and by high intra-taxon variability. Despite these limitations, CFs can help reduce misestimation in relative abundance estimates caused by variability in GCN, though their use is not yet widespread.

In addition to the 18S rRNA gene, the functional gene *rbcL* is widely used in MB analyses of phytoplankton, as it provides higher taxonomic resolution, although only for photosynthetic taxa containing the RuBisCo gene such as diatoms [44,45], enabling better discrimination of morphologically similar species or even strains of species.

Despite the growing number of studies combining LM and MB, questions remain regarding the comparability of these two methodologies and how methodological choices, such as marker selection or the application of CFs, may influence the interpretation of phytoplankton community structures. Moreover, the comparability of data across monitoring sites traditionally analyzed by LM is still poorly explored, particularly regarding whether MB may reduce site-to-site variability that might actually reflect methodological biases inherent to LM rather than true ecological differences.

Therefore, this study aims to compare the diversity and relative abundance of the phytoplankton community obtained using both traditional LM analysis and DNA MB with different markers (18S V4, V9 and *rbcL*), at three LTER sites in the NAS based on samples collected in 2019.

The combination of LM and MB has already been applied in a previous study conducted at two of the aforementioned stations, demonstrating that the integration of these two approaches provides a more comprehensive understanding of phytoplankton communities and that LM remains essential in phytoplankton monitoring, as it delivers quantitative estimates such as abundance and biomass [44,46]. For this reason, in the present study we tested the correction factors (CFs) proposed by Martin [42] with the aim of evaluating how they influence the quantitative interpretation of MB-based phytoplankton community data. By extending the same methodological framework to additional sites, we tested the hypothesis that MB, being inherently more objective and less operator-dependent than traditional LM, could reduce the differences previously observed among the three LTER sites in terms of phytoplankton community structure.

## 2. Materials and Methods

### 2.1. Study Areas and Sampling

**Figure 1:**
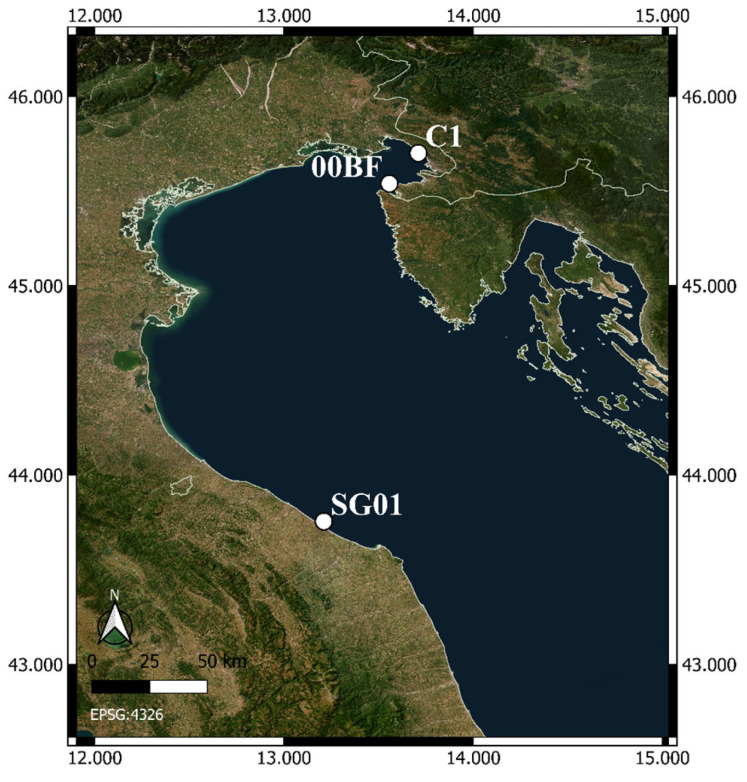
A map of the study area in the northern Adriatic Sea with the three sampling sites: C1 (Gulf of Trieste, Italy), SG01 (coastal station of the Senigallia-Susak Transect, Italy), and 00BF (Gulf of Trieste, Slovenia).

Samples were collected at three LTER sites (Figure 1)—SG01, the coastal station of the Senigallia-Susak Transect in Italy (43.755° N, 13.2105° E; https://deims.org/be8971c2-c708-4d6e-a4c7-f49fcf1623c1 accessed on 10 November 2025), C1 (45.7008° N, 13.7100° E; https://deims.org/96969205-cfdf-41d8-979f-ff881ea8dc8b accessed on 10 November 2025) and 00BF (45.540° N, 13.557° E; https://deims.org/f2ce5ae3-8873-4a8b-abad-d56d5d6da164, accessed on 10 November 2025)—located in the Gulf of Trieste, in Italy and Slovenia, respectively.

Sampling was carried out in 2019 onboard R/V Spazzamare, Sagita and Actea at C1, 00BF and SG01, respectively. The sampling months are shown in Table 1.

At all the sites, the samples were collected at the surface (0.5 m) using Niskin bottles. For LM analysis, the phytoplankton samples were stored in dark glass bottles and preserved with 0.8–2% pre-filtered and neutralized formaldehyde [47]. For MB, 5 and 2 L of seawater were filtered at sites C1 and SG01, respectively, through cellulose nitrate or acetate filters (47 mm diameter and 1.2 μm pore size, Sartorius) and stored at −80 °C and −20 °C until subsequent MB analysis. At site 00BF, 1 L of seawater was filtered in triplicate onto 0.8 μm polycarbonate filters without prefiltration, and the filters were stored at −80 °C.

### 2.2. DNA Extraction, Sequencing and Bioinformatic Analyses

The DNeasy PowerWater Kit (QIAGEN, Hilden, Germany) was used for DNA extraction following the manufacturer’s instructions. The filters were cut in half; then, each half was extracted separately and pooled after elution. A Qubit Fluorimeter (Thermo Fisher Scientific, Waltham, MA, USA) was used to assess the quantity of extracted DNA.

Three markers were considered for MB, the V4 and V9 regions of 18S rRNA and the *rbcL* chloroplast gene. Amplification was performed following the Illumina Sequencing Library Preparation protocol using 30 PCR cycles during the amplicon PCR step. Primers from Piredda [38] were used for 18S: 18S-V9F (TTGTACACACCGCCCGTCGC) and 18S-V9R (CCTTCYGCAGGTTCACCTAC) for the V9 region, and 18S-V4F (CCAGCASCYGCGGTAATTCC) and 18S-V4R (ACTTTCGTTCTTGATYRATGA) for the V4 region. For the *rbcL* region, diatom-specific primers modified from Vasselon [48] were used, i.e., 708F-DEG (AGGTGAAGYWAAAGGTTCWTAYTTAAA) and R3-DEG (CCTTCTAATTTACCWACWACWG). The PCR products were ~470 bp, ~270 bp and ~312 bp for V4, V9 and *rbcL*, respectively.

Library preparation (including IDT for Illumina UD index set D (Illumina, San Diego, CA, USA), primers with sequence complementary to overhang adapter and sample specific barcodes), 2 × 250 bp paired-end sequencing of equimolar ratios of the purified amplicon libraries on an Illumina MiSeq platform (Illumina, San Diego, CA, USA), and demultiplexing were performed at the Department of Bioscience, Biotechnology and Biopharmaceutics at Università degli Studi di Bari for the samples collected at C1 and SG1 and at BMR Genomics Srl (Padua, Italy) for the 00BF samples.

For SG1, the sample taken in May failed to amplify the V4 and *rbcL* regions, and the sample taken in June failed to amplify the *rbcL* region, thus they were not sequenced. Similarly, at 00BF, the June surface samples failed to produce enough sequencing reads and were not further processed.

For all the sites and sampling months, the amplified regions (V9 and V4 of the 18S rRNA and *rbcL*) are shown in Table 1.

Quality control and adapter detection for each 18S and *rbcL* region were assessed using FASTQC (v. 0.11.9) [49]. Primer sequences were trimmed from the demultiplexed reads using Cutadapt (v. 4.5) [50]. The resulting sequences were then processed and analyzed with the open-source platform QIIME2 (v. 2024.2) [51].

Quality filtering, denoising and pairing of the reads were performed with DADA2 [52]. The datasets from C1 and SG1 were processed independently from 00BF using the same primer removal and trimming parameters. After denoising, representative sequences were merged to allow for consistent taxonomic assignment of amplicon sequence variants (ASVs) across all the datasets using a Naive Bayes classifier [53] trained for the specific 18S rRNA gene target regions (V4 and V9) against the PR2 v. 5.0.1 [32] and for *rbcL* against R-Syst::diatom [54].

### 2.3. Phytoplankton Analysis

Phytoplankton was analyzed with an inverted microscope equipped with phase contrast following the Utermöhl method [55]. Identification and counting were performed at 400× magnification along transects or in random visual fields, based on cell abundance (to count a minimum of 200 cells). Phytoplankton cells were identified at the lowest possible taxonomic level and categorized into the main groups of diatoms, dinoflagellates (both autotrophic and heterotrophic species), coccolithophores and phytoflagellates. The latter include nanoplanktonic taxa that are mostly not identifiable by LM and include haptophytes (except coccolithophores), cryptophytes, chrysophytes, dictyochophytes, raphidophytes, chlorophytes, and euglenophytes. Possibly nano-heterotrophic taxa could have been included in this group.

### 2.4. Data and Statistical Analysis

For a consistent comparison between LM and MB, a filtering step was performed on the MB datasets using the R package Phyloseq (v.1.15.9) [56]. Specifically, phyla or classes not associated with phytoplankton (e.g., ciliates, choanoflagellates, tintinnids, and metazoans) and organelle sequences (mitochondrial and nucleomorphic) were removed from the analysis. Genus and species names were verified using the AlgaeBase database [57] to ensure the use of updated nomenclature. Additional taxonomic validation was performed through the NCBI in cases of uncertainty based on ASV-level sequences.

The MB dataset used the same classification of phytoplankton into higher taxonomic groups (diatoms, dinoflagellates, and coccolithophores) and ecological groups (phytoflagellates) as used in LM to ensure consistency between approaches.

For the analyses performed at the genus and species levels, sequences whose taxonomy ended at higher levels, e.g., classes or higher (for genera analysis) and genera or higher (for species analysis), were removed.

To avoid overestimation or biases arising from unequal sampling frequencies among approaches, all graphical representations (e.g., bar plots and Venn diagrams) and statistical analyses were performed only for the months common to the approaches considered in each analysis (Tables S1–S4).

Richness, i.e., number of genera and species, was evaluated per site and approaches and visualized using bar plots generated with the ggplot2 package [58]. The Kruskal–Wallis test was used to assess differences in phytoplankton richness among the approaches (LM, V9, and V4) within each site using the kruskal.test function in R. In case of significant differences, Dunn’s post hoc pairwise comparisons with Bonferroni correction were performed using the dunn.test function from the dunn.test package [59]. To assess the capability of *rbcL* in detecting diatom richness, the numbers of genera and species obtained with the different approaches (LM, V9, V4, and *rbcL*) were compared using the same statistical tests. In case of comparison between two variables (e.g., LM and V9 for the 00BF site), two-sample Wilcoxon tests (also known as the ‘Mann–Whitney’ test) was applied using the Wilcox.test function in the stats package [60]. The same tests were conducted across sites to compare community richness obtained using the four approaches (LM, V9, V4, and *rbcL*), assuming that filtering seawater volumes within the 1–5 L range does not significantly affect phytoplankton diversity. This assumption is supported by Pascoal et al. [61], who demonstrated that filtering different seawater volumes, ranging from 1 L up to 1000 L, does not significantly influence prokaryotic or protist diversity, regardless of the sequencing strategy.

Venn diagrams illustrating shared and unique genera and species detected by each approach (LM, V9, V4, and *rbcL*) at each site using the sampling months shared between sites were generated using the R package eulerr [62].

To test whether CFs can improve comparability between MB and LM data, the MB data were corrected using the gene copy number (GCN) CFs provided by Martin et al. [42]. The correction assumes that one sequencing read corresponds to one gene copy, making read counts proportional to the total 18S rRNA gene copies in the sample. The following group-specific CFs were used: 166 GCN cell^−1^ for diatoms, 4919 GCN cell^−1^ for dinoflagellates, and 5.23 GCN cell^−1^ for phytoflagellates and coccolithophores. To obtain the corrected relative abundances, ASV counts were divided by the corresponding CF for each group.

To examine for potential differences in phytoplankton composition in terms of genera, group-specific CFs corresponding to each genus were applied. Dominant genera were defined as those with mean relative abundances ≥1%. This threshold was applied to reduce the influence of most infrequently detected genera.

Prior to multivariate statistical analyses, a Centered Log-Ratio (CLR) transformation was applied to species relative abundance data to properly handle compositionality. A small pseudocount (1 × 10^−6^) was added to all the values to avoid issues with zeros prior to transformation. Hierarchical cluster analysis (HCA) was then conducted to assess the similarity of community composition in terms of species relative abundances across different approaches and sampling sites using Euclidean distance and the Ward.D2 linkage method. The latter was chosen among the linkage methods (ward.D2, average, complete, and single) based on the highest cophenetic correlation coefficient calculated with the cophenetic function from the R stats package, version 4.3.2. [60]. The optimal number of clusters among the samples was evaluated using three complementary approaches (the average silhouette width, the gap statistic calculated with hierarchical clustering, and the elbow method using the silhouette, fviz_nbclust, and kmeans functions, respectively, from the cluster (Maechler et al. [63]) and factoextra (Kassambara and Mundt [64]) R packages) and assessed as 3.

To visualize the species contributing most to the differences among clusters, including those detected more or less effectively by different approaches, a heatmap was generated from CLR-transformed species abundances. The plot displays the 50 most variable taxa (i.e., those with the highest standard deviation of CLR values) across all the samples, representing the main contributors to community differentiation, with values indicating deviations in relative abundance from the geometric mean of each species across all samples (from higher-than-average to lower-than-average abundance on the CLR scale).

All the analyses and graphical representations were performed and created using R software [60], version 4.3.2.

## 3. Results

### 3.1. Richness of Phytoplankton Genera and Species

A total of 329 genera and 527 species were recorded across the three sites using a combination of LM and MB (Tables S5 and S6).

**Figure 2:**
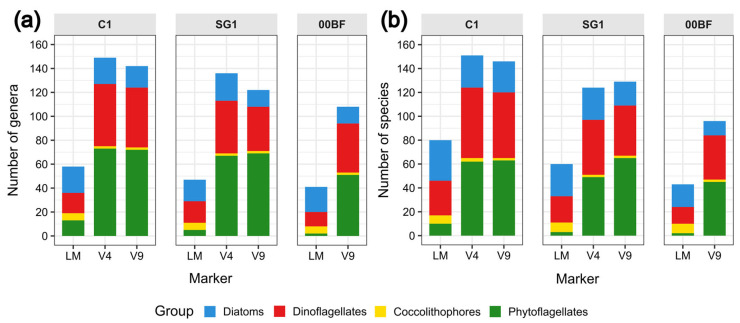
Bar plots showing number of genera (**a**) and species (**b**) of diatoms, dinoflagellates, coccolithophores, and phytoflagellates identified using LM, 18S V4, and 18S V9 and *rbcL* at three sampling sites (C1, SG1, and 00BF) during common sampling months at each site.

Looking at the number of genera and species in the whole community (Figure 2a and Figure 2b, respectively), LM analysis identified diatoms as the most species-rich group at all three sites, while MB analysis identified phytoflagellates as the most diversified group in both the V4 and V9 markers. At each site, the genus richness identified by MB analysis was higher than the LM analysis (Kruskal–Wallis test: C1 χ^2^ = 10.26, df = 2; SG1 χ^2^ = 9.57, df = 2; 00BF χ^2^ = 3.92, df = 1; all *p* < 0.05), with the V4 marker detecting the largest number of genera at C1 and SG1, and V9 at 00BF. At the species level, richness was higher in the MB data than in LM in C1 and SG1 (Kruskal–Wallis test: C1 χ^2^ = 6.23, df = 2; SG1 χ^2^ = 11.41, df = 2; all *p* < 0.05), while at 00BF it was higher, but not significant (Kruskal–Wallis test: χ^2^ = 3.58, df = 1, *p* = 0.06). At C1, the largest number of species was detected with V4, whereas at SG1 and 00BF this was observed with V9.

Richness in terms of diatom genera and species obtained with the different approaches (LM, V4, V9, and *rbcL*) is shown in Figure S1. A higher number of genera and species was found using *rbcL* compared to other approaches at sites C1 and SG1 (Kruskal–Wallis test: C1 χ^2^ = 22.61, df = 3; SG1 χ^2^ = 13.91, df = 3; all *p* < 0.01). At 00BF, the richness was also higher with *rbcL*, although a significant difference was observed only between *rbcL* and V9 (Dunn’s test: Z = 2.32, *p* = 0.03).

When comparing the richness values obtained with each approach among the sites, no significant difference was found using LM, V9, V4 and *rbcL*.

### 3.2. Diversity of Phytoplankton at the Species Level

**Figure 3:**
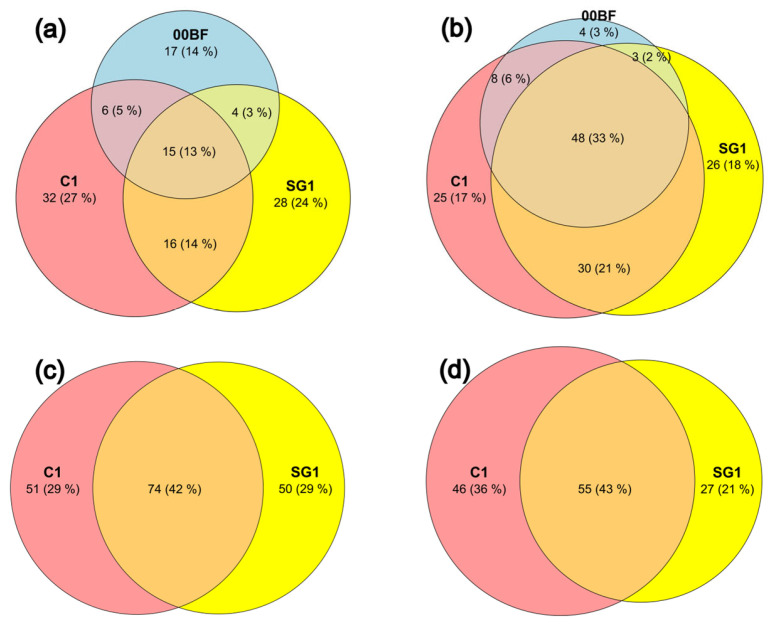
Venn diagrams showing the number and percentage of shared and unique species among the three sites (C1, SG1, and 00BF) for LM (**a**), V9 (**b**), V4 (**c**) and *rbcL* (**d**), during the respective common sampling months.

The LM analysis identified a total of 118 species, 13% of which were shared among the sites (Figure 3a). Many species were found uniquely in each site, with the highest percentage in C1 (27%), followed by SG1 (24%) and 00BF (14%). The greatest overlap is observed between C1 and SG1, sharing 27% of the species.

MB detected a higher number of species, with 144 identified by the V9 marker and 175 by the V4 marker (Figure 3b and Figure 3c, respectively). In both cases, a high number of species shared among all the sites was observed (33% for V9 and 42% for V4), although with local differences: SG1 shows the highest number of unique species with V9, while C1 with V4. With the V9 marker, most of the sharing occurs between C1 and SG1 (54%). The *rbcL* marker, for which only species belonging to the diatom group were considered, detected a total of 131 species, 43% of which are shared between C1 and SG1 (Figure 3d).

### 3.3. Community Composition and Correction Factors

**Figure 4:**
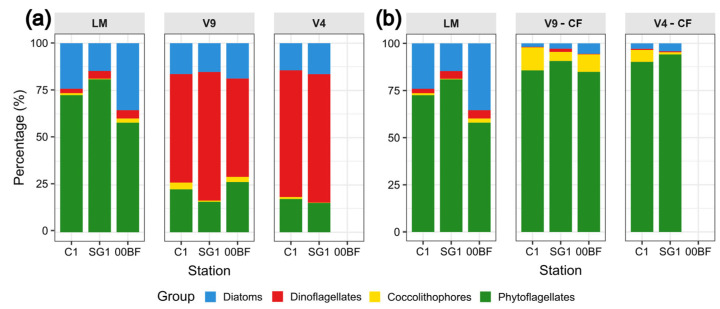
Bar plots representing the relative abundances of main phytoplankton groups obtained using the three approaches without (**a**) and with (**b**) the use of correction factors for each site during the common sampling months.

Relative abundances of phytoplankton groups as obtained by the three methodological approaches is shown in Figure 4. Without the application of the CFs (Figure 4a), the community structure obtained through LM markedly differed from that derived from MB at all the study sites. The LM data indicated a dominance of phytoflagellates across all the stations (ranging from 58 to 81%), whereas the MB data showed a predominance of dinoflagellates (52–68% and 67–68% with V9 and V4, respectively). The diatoms’ relative abundances were similar between the approaches, ranging from 15 to 35% with LM and from 14 to 19% with MB. Coccolithophores were consistently detected at low relative abundances across all the approaches, with values below 4% in both the LM and MB data.

After applying CFs to the MB data (Figure 4b), phytoflagellates became the dominant group at all the sites (85–91% with the V9 marker and 90–94% with V4), bringing the values closer to the LM estimates (58–81%). Coccolithophores emerged as the second most abundant group with marker V9 in all the three stations (5–12%) and with V4 in C1 (6%). Diatoms showed consistently low relative abundances (<6%) in all the MB samples, differing from the higher values observed by LM (15–35%). Dinoflagellates became the least represented group in the MB data after the application of CF, accounting for less than 2% across all the sites.

The dominant genera identified using LM and MB, with and without CF application, are shown in Figure S2. LM revealed the diatoms *Chaetoceros* (61% in C1), *Nitzschia* (54% in SG1) and *Cyclotella* (37% in 00BF) as the dominant genera. Without the application of CFs, the dominant genera with the V9 marker were *Chaetoceros* (16% in C1 and 52% in 00BF) and the dinoflagellate *Alexandrium* (42% in SG1), while with the V4 marker, the dominant genera were the dinoflagellates *Heterocapsa* (23% in C1) and *Noctiluca* (21% in SG1). After applying CFs on the MB data, the dominant genera with the V9 marker were the prasinophyte *Micromonas* (31% in SG1) and the haptophyte *Chrysochromulina* (21% in C1 and 20% in 00BF), while with the V4 marker it was *Micromonas* (24% in C1 and SG1).

### 3.4. Cluster Analysis

**Figure 5:**
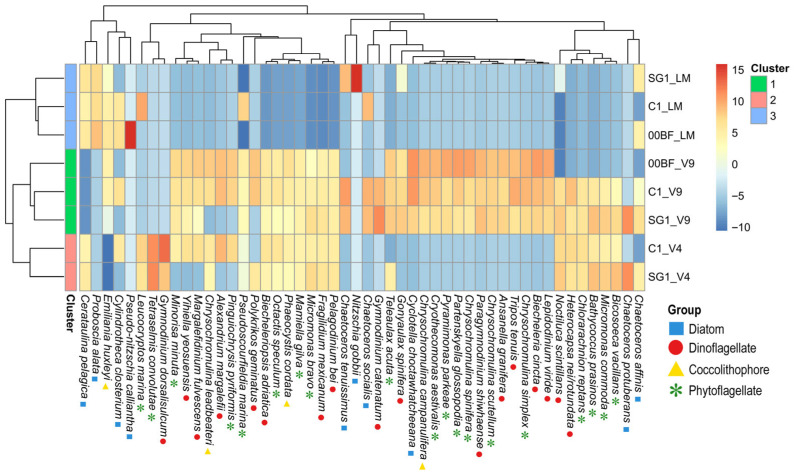
Heatmap of 50 most variable species showing CLR-transformed relative abundances across sites (C1, SG1, and 00BF) and molecular markers (LM, V9, V4), including only samples collected in common months. Colors indicate deviations in relative abundance from geometric mean of each species across all samples (CLR-transformed values), with red corresponding higher-than-average abundance, white corresponding to abundances close to geometric mean (i.e., near-zero CLR values, which can also include samples with zero observed counts), and blue corresponding lower-than-average abundance.

Cluster analysis performed on the CLR-transformed community data revealed three groups, each characterized by samples related to the same approaches (Figure S3 and Figure 5). In particular, the species detected using the LM samples grouped together in a distinct cluster at lower dissimilarity levels (height < 100), while those obtained with the V9 and V4 markers were organized into two separate groups at higher dissimilarity levels (height ≈ 150).

A heatmap of the top 50 most variable species (CLR-transformed abundances) is shown in Figure 5. Although with some differences among the stations, in the LM cluster a few diatoms, *Proboscia alata*, *Pseudo-nitzschia calliantha*, and *Nitzschia gobbii* exhibited high CLR values (>5), whereas the same species were present at lower abundances or not detected in the V4 and V9 MB clusters. In contrast, several species displayed high CLR values only in the MB clusters (V4 and/or V9), ranging from +2 to +10. These included diatoms (e.g., *Chaetoceros protuberans* and *Cyclotella choctawhatcheeana*), dinoflagellates (e.g., *Biecheleriopsis adriatica*, *Biecheleria cincta*, *Pelagodinium bei*, *Margalefidinium fulvescens*, *Alexandrium margalefii*, *Gonyaulax spinifera*, *Fragilidium mexicanum*, *Ansanella granifera*, *Gymnodinium catenatum*, *G. dorsalisulcum*, *Noctiluca scintillans*, *Yihiella yeosuensis*, *and Polykrikos geminatus*), and various phytoflagellates (e.g., *Minorisa minuta*, *Micromonas commoda*, *M. bravo*, *Octactis speculum*, *Bathycoccus prasinos*, *Mamiella gilva*, *Tetraselmis convolutae*, *Cryothecomonas aestivalis*, *Pyramimonas parkeae*, *Chrysochromulina scutellum*, *C. simplex*, *C. spinifera*, *C. leadbeateri*, *Partenskyella glossopodia*, *Phaeocystis cordata* and *Teleaulax acuta*).

A subset of taxa, including *Chaetoceros socialis*, *C. tenuissimus*, *C. affinis*, *Cylindrotheca closterium* and *Leucocryptos marina*, displayed positive CLR values (>2) across both the LM and MB samples, indicating relatively stable detection within the approaches, although with differences among the stations.

## 4. Discussion

In this work, we applied a multi-marker MB approach (targeting the V4 and V9 regions of the 18S rRNA gene and the *rbcL* gene) in combination with traditional LM to investigate phytoplankton diversity and community composition at three LTER sites in the northern Adriatic Sea, revealing marked differences in taxonomic richness, community composition, and relative abundance estimates between the two approaches.

The phytoplankton communities of the study sites have been intensively studied through long-term microscopic surveys since 1986 at station C1, since 1988 at SG1, and since 1990 at 00BF. Over these decades, monitoring has documented remarkable taxonomic richness, with 310 taxa reported at C1 (data not published), 593 taxa at SG1 [13,65], and 130 taxa at station 00BF [66]. In the present work, the combination of V4, V9, and *rbcL* markers led to an increase in detected diversity, thanks to the identification of previously unreported taxa. In this study, 259 genera and 416 species not previously reported in the study areas were identified across the three sites. These results confirm that MB may reveal higher diversity compared to traditional LM [38,44,46,67,68,69,70].

In the study areas, the increased richness detected by MB compared to LM was not evenly distributed among the phytoplankton groups but was mainly associated with dinoflagellates and phytoflagellates rather than diatoms and coccolithophores. This observation was further supported by the heatmap derived from the cluster analysis (approach considering species and their relative abundances), which highlighted the differential detection of major phytoplankton groups between the two approaches; most species detected exclusively (or with high relative abundances) by LM were diatoms, while those by MB were mainly dinoflagellates and phytoflagellates. Several factors may explain these results. First, the fixative traditionally used at the three sites, i.e., formaldehyde, is particularly suitable for preserving diatoms, which may have contributed to the long-term success of microscopic identification of this group [71,72,73], whereas it compromises the identification of naked dinoflagellates and phytoflagellates [47,73,74,75,76,77]. Additionally, the experience of taxonomists in these regions has a strong tradition in diatom studies [78,79], which further enhances their representation in the dataset. Regarding coccolithophores, taxa are often underrepresented in MB analyses due to a suboptimal primer performance for haptophytes [80] and to their limited presence in reference databases, a consequence of the difficulties associated with their cultivation. Indeed, most of the coccolithophore species identified by LM (*Acanthoica quattrospina*, *Calciosolenia brasiliensis*, *C. murrayi*, *Ophiaster hydroideus*, *Rhabdosphaera clavigera*, *Calyptrosphaera oblonga*, *Michaelsarsia adriatica*, *M. elegans*, and *Syracosphaera histrica*) were found to be absent from the reference databases, and therefore were not detected by MB. This highlights the importance of expanding databases with high-quality sequences obtained from well-characterized monoclonal cultures [38,44,67,68,81].

During the study period, LM revealed some species that were unique to each sampling site and others shared among all the sites. However, the number of species shared among the sites approximately doubled using MB, suggesting that this approach can indeed reduce differences among sites. In particular, although different pore-size filters (1.2 and 0.8 µm)—a feature most important in comparing MB datasets [61]—were used within the Gulf of Trieste (C1 and 00BF, respectively), MB still revealed only a limited number of taxa unique to each site. However, inter-site differences were observed, and these discrepancies may reflect ecological differences, sampling variability or bioinformatic assignment errors [21,81,82,83,84]. Moreover, methodological differences among the sites (filtered volumes, pore sizes and storage temperatures) could potentially affect the MB outputs. However, their impact on inter-site comparability is likely limited, given the narrow range of protocols applied and evidence that such variations do not substantially influence diversity estimates [61].

The idea that MB may reduce the differences between sites not only due to true ecological variation, but also to methodological biases inherent to LM, is further supported by the cluster analysis, which grouped the samples primarily according to the methodological approach, including the MB markers, rather than by site. This pattern suggests that methodological differences exceed ecological variability, as LM and MB capture partially distinct components of the phytoplankton community. LM primarily detects morphologically identifiable and often larger taxa, such as diatoms, whereas MB is capable to detect small, fragile, or morphologically cryptic taxa, including many dinoflagellates and phytoflagellates. In addition, methodological biases related to sample preservation, primer specificity, gene copy number variation, and taxonomic reference database completeness further shape the detected community structure. Moreover, data processing choices and transformations inherent to MB data may also amplify these differences, reinforcing method-driven clustering patterns.

The composition of the phytoplankton community varied substantially depending on the approach used (LM, V4, V9, and *rbcL*), even in terms of taxonomic resolution. Indeed, V9 detected the largest number of species at SG1, and V4 was more effective at C1, but this comparison excludes 00BF, as only the V9 marker was applied. Considering only diatoms, the *rbcL* marker, specifically designed for diatoms and supported by a more complete and curated reference database [85], detected a consistently higher number of genera and species compared to both LM and 18S rRNA markers, providing higher diversity compared to the other markers and LM (e.g., [44,86,87]).

The methodological differences were also reflected on the relative abundance estimates; LM indicated a predominance of phytoflagellates, followed by diatoms, dinoflagellates and coccolithophores, while MB pointed to higher relative abundances of dinoflagellates, followed by coccolithophores, diatoms, and phytoflagellates. These differences are likely due to the lack of a direct correspondence between cell numbers and sequence counts for certain groups, which can lead to misestimations. This can be explained considering that cells with a high DNA content, such as those of dinoflagellates [88,89,90], tend to generate higher read numbers during sequencing. Indeed, after applying CFs, the relative abundances obtained by MB showed greater similarity to those obtained by LM, with an increase in phytoflagellates and a decrease in dinoflagellates. However, the CFs appear to underestimate diatom relative abundances. This could be explained by the fact that diatoms have highly variable biovolumes, ranging from 10^1^ to 10^9^ μm^3^ [91], and as the biovolume increases, so do the copies of the 18S rRNA gene [92]. Such variability makes it challenging to develop and apply a CF accurate for all diatoms, and genus-level CFs may be more appropriate [93].

It is worth noting that the CF application actually adjusts relative abundance and does not convert them into absolute abundances. Their main role is to smooth out the differences between the molecular and traditional approaches, though it does not fully resolve them. Therefore, even after the application of correction factors, MB-derived abundance estimates should be considered as semi-quantitative and interpreted with caution when compared with LM-based cell counts. Indeed, the analyses of dominant genera show that even after applying the CFs, their results remain highly dissimilar between LM and MB and between V4 and V9. This suggests that to achieve the most accurate correction of MB values, a specific CF should be calculated and applied for each taxon.

## 5. Conclusions

Understanding phytoplankton diversity and community composition is essential for assessing ecosystem health and monitoring environmental change, which requires accurate species identification. In this study, the combination of multi-marker MB (targeting the V4 and V9 regions of the 18S rRNA gene and the *rbcL* gene) with traditional LM revealed that MB detects increased phytoplankton richness compared to LM at three LTER sites in the northern Adriatic Sea, particularly for dinoflagellates and phytoflagellates. The use of alternative fixatives, such as Lugol alongside formalin, could improve the LM detection of delicate taxa like dinoflagellates, while the expansion of reference databases through cultivation and sequencing of previously unrepresented species would enhance the taxonomic resolution of MB. Overall, 329 genera and 527 species were recorded by integrating the two methods, underscoring the complementary strengths of both the approaches. Metabarcoding also increased the proportion of species shared among the sites, improving inter-site comparability.

Despite variations among the sampling sites and local environmental conditions, the overall similarity in phytoplankton composition across the northern Adriatic indicates a shared regional pool of species. Although some ecological variation was certainly present, the differences observed among the sites were primarily attributed to the methodological choices, such as the use of MB versus LM and the selection of molecular markers.

Differences in relative abundance estimates between LM and MB further highlight the need for refined correction factors (CFs). Although CFs can partially reconcile the two methods, as occurs for dinoflagellates, for which overestimation is reduced after the CF application, they remain insufficiently effective for the other groups. Developing taxon-specific CFs and expanding curated barcode databases are therefore critical steps for improving the quantitative reliability of molecular assessments.

Overall, this study demonstrates that integrating molecular and morphological approaches provides a more comprehensive framework for long-term phytoplankton monitoring. This integrated strategy improves biodiversity assessment and strengthens data comparability across monitoring sites. Future efforts should focus on protocol harmonization across sites, taxon-specific quantitative calibration, and continued improvement of reference libraries to enhance data comparability within monitoring networks such as LTER.

## Figures and Tables

**Table 1:** Monthly sampling and type of analysis in 2019. Brackets ([ ]) indicate samples that were excluded from analysis due to failed amplification or sequencing.

	C1				SG1				00BF		
	LM	18S V4	18S V9	*rbcL*	LM	18S V4	18S V9	*rbcL*	LM	18S V9	*rbcL*
January	X										
February	X	X	X	X	X	X	X	X			
March	X	X	X	X	X	X	X	X	X	X	
April	X	X	X	X	X	X	X	X	X		
May	X	X	X	X	X	[X]	X	[X]	X	X	
June	X	X	X	X	X	X	X	[X]	X	[X]	[X]
July	X	X	X	X	X	X	X	X	X	X	
August	X	X	X	X					X	X	
September	X	X	X	X	X	X	X	X	X	X	X
October	X	X	X	X	X	X	X	X	X		
November	X	X	X	X					X	X	X
December	X	X	X	X					X	X	X

## Data Availability

Raw sequence data generated in this study, as well as the microscopy data and related metadata, can be obtained from the corresponding author upon reasonable request.

## Supplementary Materials

The following supporting information can be downloaded at: https://www.mdpi.com/article/10.3390/biology15060487/s1, Figure S1: Bar plots showing the number of genera (a) and species (b) of diatoms identified using LM, 18S V4, 18S V9, and *rbcL* at three sampling sites, during the common sampling months at each site; Figure S2: Percentages of dominant genera at each site, during the common sampling months at each site, obtained using the three approaches: LM (violet), V9 (green), and V4 (orange), before (a) and after (b) the application of CFs. Figure S3: Hierarchical dendrogram of phytoplankton species from the three sampling sites, constructed using CLR-transformed data and considering only the months of sampling in common; Table S1: Months considered for estimation and graphical representation of richness in each site; Table S2: Months considered for the construction of the Venn diagrams; Table S3: Months considered for the representations of relative abundances in bar plots, with and without the application of correction factors; Table S4: Months considering in the hierarchical clustering analysis; Table S5: List of genera recorded in the three sites combining LM and MB. Table S6. List of species recorded in the three sites combining LM and MB.
