# Supplementary material for: Phytoplankton Diversity in the Northern Adriatic Sea: Insights and Inconsistencies from Microscopy and Metabarcoding
Source: Biology (Basel). 2026 Mar 19;15(6):487. doi: 10.3390/biology15060487 (PMC13023596; doi:10.3390/biology15060487)
Supplement: Supplementary file 1 [file biology-15-00487-s001.zip › Supplementary-Figures.pdf]

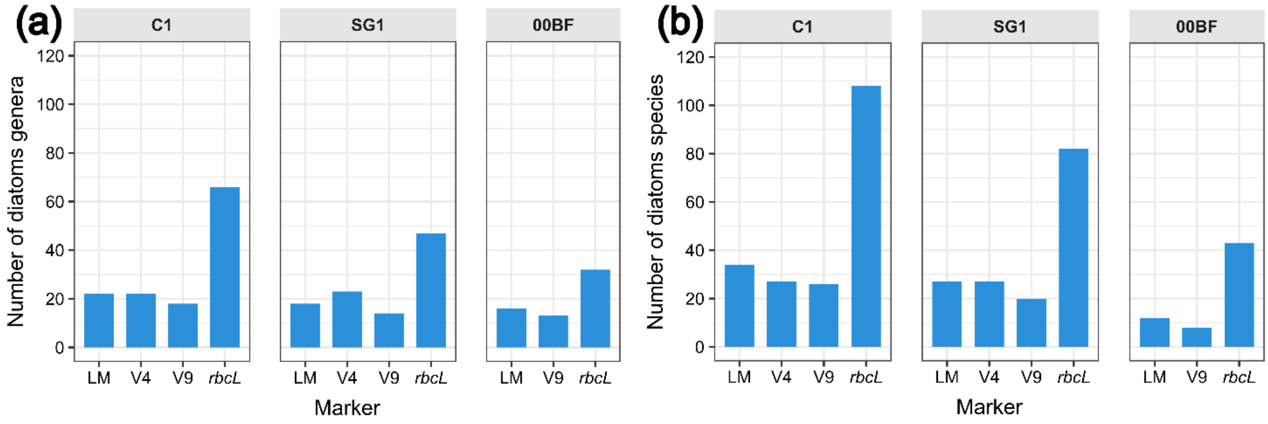

Figure S1. Bar plots showing the number of genera (a) and species (b) of diatoms identified using LM, 18S V4, 18S V9, and *rbcL* at three sampling sites, during the common sampling months at each site.

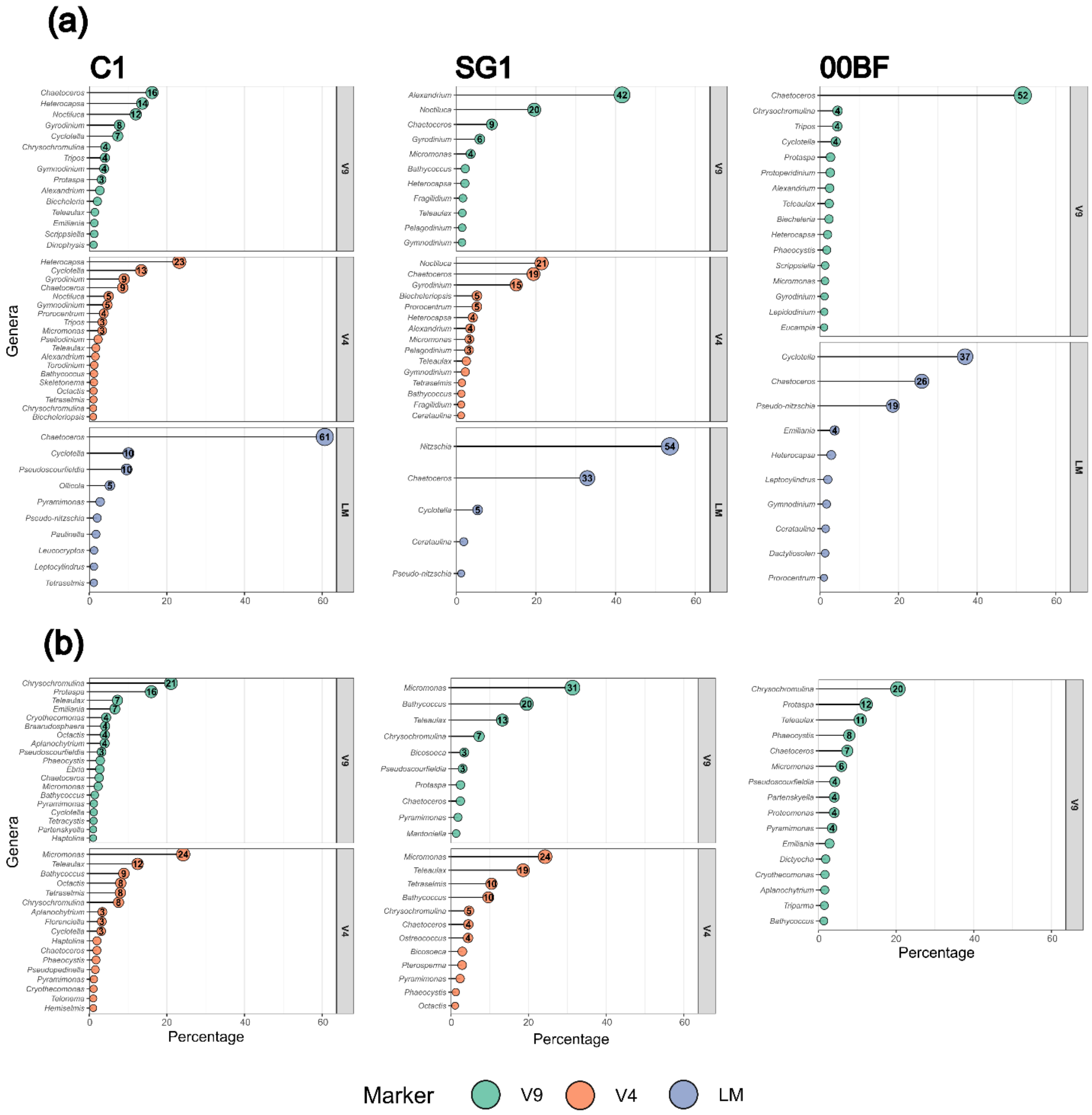

Figure S2. Percentages of dominant genera at each site, during the common sampling months at each site, obtained using the three approaches: LM (violet), V9 (green), and V4 (orange), before (a) and after (b) the application of CFs.

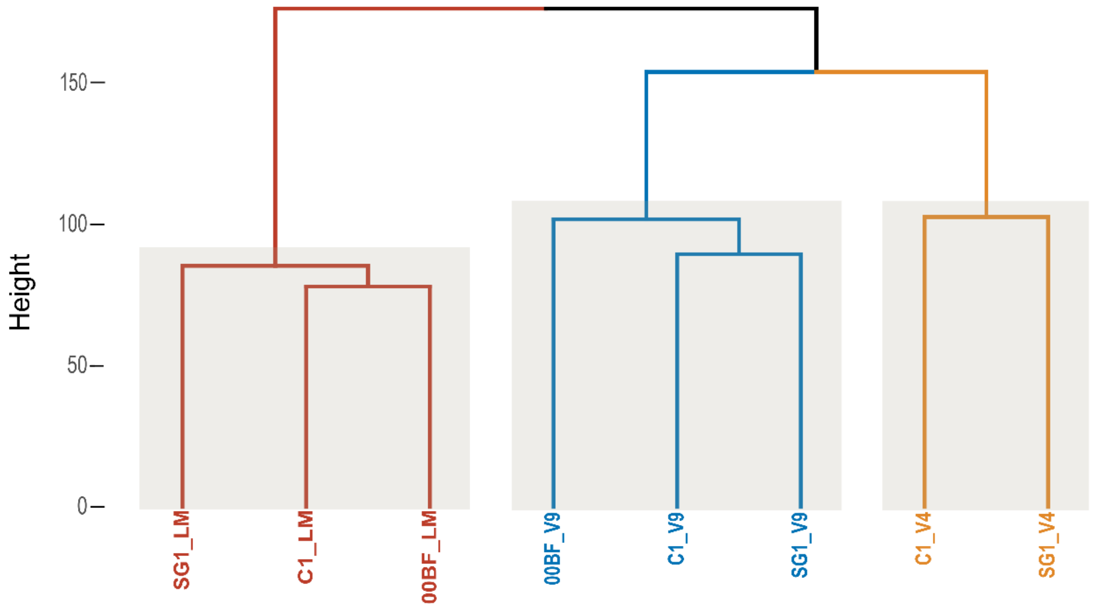

Figure S3. Hierarchical dendrogram of phytoplankton species from the three sampling sites, constructed using CLR-transformed data and considering only the months of sampling in common.
