# Supplementary material for: Phytoplankton Diversity in the Northern Adriatic Sea: Insights and Inconsistencies from Microscopy and Metabarcoding
Source: Biology (Basel). 2026 Mar 19;15(6):487. doi: 10.3390/biology15060487 (PMC13023596; doi:10.3390/biology15060487)
Supplement: Supplementary file 1 [file biology-15-00487-s001.zip › Supplementary-Tables.pdf]

Table S1. Months considered for estimation and graphical representation of richness in each site.

	C1				SG1				00BF		
	**LM**	**18S V4**	**18S V9**	***rbcL***	**LM**	**18S V4**	**18S V9**	***rbcL***	**18S LM**	**18S V9**	***rbcL***
**January**											
**February**	X	X	X	X	X	X	X	X			
**March**	X	X	X	X	X	X	X	X			
**April**	X	X	X	X	X	X	X	X			
**May**	X	X	X	X							
**June**	X	X	X	X							
**July**	X	X	X	X	X	X	X	X			
**August**	X	X	X	X							
**September**	X	X	X	X	X	X	X	X	X	X	X
**October**	X	X	X	X	X	X	X	X			
**November**	X	X	X	X					X	X	X
**December**	X	X	X	X					X	X	X

Table S2. Months considered for the construction of the Venn diagrams.

	**LM**	**V9**	**V4**	***rbcL***
	**C1-SG1-00BF**	**C1-SG1-00BF**	**C1-SG1**	**C1-SG1**
**January**				
**February**			X	X
**March**	X	X	X	X
**April**	X		X	X
**May**	X	X		
**June**	X		X	
**July**	X	X	X	X
**August**				
**September**	X	X	X	X
**October**	X		X	X
**November**				
**December**				

Table S3. Months considered for the representations of relative abundances in bar plots, with and without the application of correction factors.

	**LM**	**V9**	**V4**
	**C1-SG1-00BF**	**C1-SG1-00BF**	**C1-SG1**
**January**			
**February**			X
**March**	X	X	X
**April**	X		X

**May**	X	X	
**June**	X		X
**July**	X	X	X
**August**			
**September**	X	X	X
**October**	X		X
**November**			
**December**			

Table S4. Months considering in the hierarchical clustering analysis.

	**LM-V9-V4**
	**C1-SG1-00BF**
**January**	
**February**	
**March**	X
**April**	
**May**	
**June**	
**July**	X
**August**	
**September**	X
**October**	
**November**	
**December**	

Table S5. List of genera recorded in the three sites combining LM and MB.

		C1				SG1				00BF		
**Group**	**Genus**	**LM**	**V9**	**V4**	***rbcL***	**LM**	**V9**	**V4**	***rbcL***	**LM**	**V9**	***rbcL***
Coccolithophores	*Acanthoica*	X										
Coccolithophores	*Algirosphaera*			X				X				
Coccolithophores	*Braarudosphaera*		X				X				X	
Coccolithophores	*Calciosolenia*	X				X				X		
Coccolithophores	*Calyptrosphaera*					X				X		
Coccolithophores	*Emiliania*	X	X			X	X			X	X	
Coccolithophores	*Michaelsarsia*					X						
Coccolithophores	*Ophiaster*	X								X		
Coccolithophores	*Rhabdosphaera*	X				X				X		
Coccolithophores	*Syracosphaera*	X		X		X		X		X		
Diatoms	*Achnanthidium*				X				X			
Diatoms	*Adlafia*								X			
Diatoms	*Amphora*				X	X		X	X	X		

Diatoms	*Arcocellulus*								X			
Diatoms	*Asterionellopsis*	X			X				X			
Diatoms	*Asteromphalus*			X	X	X		X	X	X		X
Diatoms	*Bacillaria*				X							
Diatoms	*Bacteriastrum*	X	X	X		X				X		
Diatoms	*Berkeleya*				X				X			
Diatoms	*Biremis*				X							
Diatoms	*Caloneis*				X							
Diatoms	*Ceratanaulus*						X					
Diatoms	*Cerataulina*	X		X	X	X		X	X	X		X
Diatoms	*Chaetoceros*	X	X	X	X	X	X	X	X	X	X	X
Diatoms	*Climaconeis*				X							X
Diatoms	*Cocconeis*				X							X
Diatoms	*Conticribra*		X		X							
Diatoms	*Coronia*				X							
Diatoms	*Coscinodiscus*		X	X	X				X	X	X	X
Diatoms	*Craspedostauros*				X							
Diatoms	*Craticula*								X			
Diatoms	*Cyclotella*	X	X	X	X	X	X	X	X	X	X	X
Diatoms	*Cylindrotheca*	X	X	X	X	X	X		X	X		X
Diatoms	*Cymbella*								X		X	
Diatoms	*Dactyliosolen*	X				X				X		
Diatoms	*Diatoma*								X			
Diatoms	*Diploneis*	X			X				X	X		
Diatoms	*Ditylum*	X			X		X		X			X
Diatoms	*Encyonema*							X	X			
Diatoms	*Entomoneis*				X							X
Diatoms	*Epithemia*				X							
Diatoms	*Eucampia*	X	X		X		X		X	X	X	
Diatoms	*Eunotia*				X							
Diatoms	*Extubocellulus*				X							
Diatoms	*Fallacia*				X							
Diatoms	*Fistulifera*							X	X			
Diatoms	*Fragilaria*				X							
Diatoms	*Fragilariopsis*			X							X	
Diatoms	*Gomphonema*								X			
Diatoms	*Guinardia*	X		X	X	X		X	X	X		X
Diatoms	*Gyrosigma*				X				X			
Diatoms	*Halamphora*				X				X			X
Diatoms	*Haslea*	X			X							X
Diatoms	*Hemiaulus*	X			X				X	X		X
Diatoms	*Hyalodiscus*				X							
Diatoms	*Lauderia*		X		X		X		X		X	
Diatoms	*Leptocylindrus*	X	X	X		X	X	X		X	X	
Diatoms	*Licmophora*	X			X				X	X		X
Diatoms	*Lithodesmium*		X									
Diatoms	*Mayamaea*				X		X		X			

Diatoms	*Melosira*	X					X		X			
Diatoms	*Meridion*				X							
Diatoms	*Minidiscus*		X	X	X			X	X		X	X
Diatoms	*Minutocellus*		X	X	X		X	X	X			X
Diatoms	*Navicula*		X	X	X		X	X	X			X
Diatoms	*Nitzschia*			X	X	X	X	X	X	X		X
Diatoms	*Opephora*				X							
Diatoms	*Palmerina*				X							
Diatoms	*Papiliocellulus*			X	X			X	X		X	X
Diatoms	*Paralia*				X				X			X
Diatoms	*Petrodictyon*				X							X
Diatoms	*Pinnularia*				X				X			X
Diatoms	*Planothidium*				X							
Diatoms	*Pleurosigma*	X	X	X	X	X	X	X	X	X	X	X
Diatoms	*Porosira*		X									
Diatoms	*Proboscia*	X		X		X		X		X		
Diatoms	*Psammodictyon*				X							X
Diatoms	*Pseudo-nitzschia*	X	X	X	X	X	X	X	X	X	X	X
Diatoms	*Pseudosolenia*	X			X			X	X			
Diatoms	*Ralfsiella*								X			
Diatoms	*Rhizosolenia*	X		X	X			X	X	X		X
Diatoms	*Rossithidium*				X							
Diatoms	*Sellaphora*				X							
Diatoms	*Seminavis*				X							
Diatoms	*Shionodiscus*				X							
Diatoms	*Skeletonema*	X	X	X	X	X	X	X	X	X	X	X
Diatoms	*Staurosira*				X							
Diatoms	*Striatella*				X				X			
Diatoms	*Surirella*								X			
Diatoms	*Synedra*					X						
Diatoms	*Tabularia*				X			X				
Diatoms	*Talaroneis*				X							X
Diatoms	*Tenuicylindrus*						X					
Diatoms	*Thalassionema*	X		X	X	X		X	X	X		X
Diatoms	*Thalassiosira*	X	X	X	X	X	X	X	X	X	X	X
Diatoms	*Toxarium*				X							X
Diatoms	*Tryblionella*				X							
Diatoms	*Ulnaria*								X			
Dinoflagellates	*Akashiwo*					X	X	X				
Dinoflagellates	*Alexandrium*	X	X	X		X	X	X		X	X	
Dinoflagellates	*Amoebophrya*		X	X			X	X			X	
Dinoflagellates	*Amylax*			X								
Dinoflagellates	*Ansanella*		X	X			X	X			X	
Dinoflagellates	*Archaeperidinium*						X	X				
Dinoflagellates	*Ataxiodinium*			X								
Dinoflagellates	*Azadinium*		X	X			X	X			X	
Dinoflagellates	*Balechina*			X								

Dinoflagellates	*Biecheleria*		X	X			X	X			X	
Dinoflagellates	*Biecheleriopsis*		X	X			X	X			X	
Dinoflagellates	*Bispinodinium*										X	
Dinoflagellates	*Blastodinium*		X	X			X	X			X	
Dinoflagellates	*Blixaea*							X				
Dinoflagellates	*Ceratocorys*										X	
Dinoflagellates	*Ceratoperidinium*	X										
Dinoflagellates	*Chytriodinium*		X	X			X				X	
Dinoflagellates	*Cladocopium*		X									
Dinoflagellates	*Cochlodinium*					X						
Dinoflagellates	*Cucumeridinium*			X								
Dinoflagellates	*Dinophysis*	X	X	X		X				X	X	
Dinoflagellates	*Diplopsalis*	X				X				X		
Dinoflagellates	*Durinskia*		X	X								
Dinoflagellates	*Ellobiopsis*		X	X				X			X	
Dinoflagellates	*Euduboscquella*		X	X			X	X			X	
Dinoflagellates	*Fragilidium*		X	X			X	X			X	
Dinoflagellates	*Gonyaulax*	X	X	X		X	X	X		X	X	
Dinoflagellates	*Grammatodinium*							X				
Dinoflagellates	*Gymnodinium*		X	X		X	X	X		X	X	
Dinoflagellates	*Gymnoxanthella*		X				X					
Dinoflagellates	*Gyrodiniellum*		X				X				X	
Dinoflagellates	*Gyrodinium*	X	X	X			X	X		X	X	
Dinoflagellates	*Heterocapsa*		X	X			X	X		X	X	
Dinoflagellates	*Islandinium*		X	X			X	X			X	
Dinoflagellates	*Karenia*		X			X	X	X			X	
Dinoflagellates	*Karlodinium*		X	X			X	X			X	
Dinoflagellates	*Kofoidinium*	X		X		X		X				
Dinoflagellates	*Lepidodinium*		X	X			X	X			X	
Dinoflagellates	*Lessardia*	X						X				
Dinoflagellates	*Levanderina*						X	X				
Dinoflagellates	*Lingulaulax*		X	X		X	X				X	
Dinoflagellates	*Luciella*		X									
Dinoflagellates	*Margalefidinium*		X	X			X	X			X	
Dinoflagellates	*Noctiluca*	X	X	X		X	X	X			X	
Dinoflagellates	*Nusuttodinium*		X									
Dinoflagellates	*Ornithocercus*		X									
Dinoflagellates	*Ostreopsis*		X	X								
Dinoflagellates	*Oxyphysis*					X						
Dinoflagellates	*Oxytoxum*	X				X				X		
Dinoflagellates	*Paradinium*		X									
Dinoflagellates	*Paragymnodinium*		X				X				X	
Dinoflagellates	*Pararosarium*			X								
Dinoflagellates	*Paulsenella*		X				X				X	
Dinoflagellates	*Pelagodinium*		X	X			X	X			X	
Dinoflagellates	*Pentapharsodinium*										X	
Dinoflagellates	*Phalacroma*		X	X		X	X	X			X	

Dinoflagellates	*Podolampas*	X										
Dinoflagellates	*Polykrikos*		X	X			X	X			X	
Dinoflagellates	*Posoniella*		X									
Dinoflagellates	*Prorocentrum*	X	X	X		X	X	X		X	X	
Dinoflagellates	*Proterythropsis*			X				X				
Dinoflagellates	*Protoceratium*	X	X	X		X	X	X				
Dinoflagellates	*Protoperidinium*	X	X	X		X	X	X		X	X	
Dinoflagellates	*Pseliodinium*			X				X				
Dinoflagellates	*Pyrocystis*		X	X								
Dinoflagellates	*Pyrophacus*							X				
Dinoflagellates	*Qia*			X								
Dinoflagellates	*Schuettiella*		X	X							X	
Dinoflagellates	*Scrippsiella*		X	X		X	X	X		X	X	
Dinoflagellates	*Spatulodinium*			X				X				
Dinoflagellates	*Spiniferodinium*							X				
Dinoflagellates	*Stoeckeria*		X	X							X	
Dinoflagellates	*Syltodinium*			X				X				
Dinoflagellates	*Symbiodinium*			X								
Dinoflagellates	*Torodinium*	X		X				X				
Dinoflagellates	*Triadinium*	X	X	X			X				X	
Dinoflagellates	*Tripos*	X	X	X		X	X	X		X	X	
Dinoflagellates	*Wangodinium*			X				X				
Dinoflagellates	*Warnowia*		X	X			X	X			X	
Dinoflagellates	*Yihiella*		X	X			X	X			X	
Dinoflagellates	*Zooxanthella*		X									
Phytoflagellates	*Abollifer*			X								
Phytoflagellates	*Allobodo*		X									
Phytoflagellates	*Allovahlkampfia*						X					
Phytoflagellates	*Alphamonas*		X									
Phytoflagellates	*Apedinella*	X		X			X	X				
Phytoflagellates	*Aplanochytrium*		X	X			X	X			X	
Phytoflagellates	*Apusomonas*							X				
Phytoflagellates	*Aureococcus*		X	X			X	X			X	
Phytoflagellates	*Baffinella*		X									
Phytoflagellates	*Bathycoccus*		X	X			X	X			X	
Phytoflagellates	*Bicosoeca*		X	X			X	X			X	
Phytoflagellates	*Bicosta*			X				X				
Phytoflagellates	*Bigelowiella*						X				X	
Phytoflagellates	*Botuliforma*		X				X					
Phytoflagellates	*Cafeteria*										X	
Phytoflagellates	*Calliacantha*		X	X			X					
Phytoflagellates	*Calycomonas*					X						
Phytoflagellates	*Cercomonas*		X				X					
Phytoflagellates	*Chattonella*						X	X				
Phytoflagellates	*Chlamydomonas*		X	X				X			X	
Phytoflagellates	*Chlorarachnion*		X	X			X	X			X	
Phytoflagellates	*Chlorochytrium*						X					

Phytoflagellates	*Chlorococcum*							X				
Phytoflagellates	*Chloroidium*						X	X				
Phytoflagellates	*Chloroparvula*		X	X			X	X			X	
Phytoflagellates	*Chrysochromulina*		X	X			X	X			X	
Phytoflagellates	*Chrysolepidomonas*			X				X				
Phytoflagellates	*Chrysowaernella*			X								
Phytoflagellates	*Ciliophrys*		X								X	
Phytoflagellates	*Colpodella*		X	X			X	X			X	
Phytoflagellates	*Commation*	X										
Phytoflagellates	*Cryothecomonas*		X	X			X	X			X	
Phytoflagellates	*Cymbomonas*		X	X			X	X				
Phytoflagellates	*Desmodesmus*						X	X				
Phytoflagellates	*Developayella*						X					
Phytoflagellates	*Dicrateria*			X								
Phytoflagellates	*Dictyocha*	X		X		X		X			X	
Phytoflagellates	*Dinobryon*	X			X	X			X			X
Phytoflagellates	*Diplonema*		X				X					
Phytoflagellates	*Discomonas*			X								
Phytoflagellates	*Dolichomastix*			X				X				
Phytoflagellates	*Ebria*		X				X				X	
Phytoflagellates	*Euglena*					X						
Phytoflagellates	*Euglypha*		X				X				X	
Phytoflagellates	*Eutreptiella*		X									
Phytoflagellates	*Fibrocapsa*						X	X				
Phytoflagellates	*Fibrophrys*			X				X				
Phytoflagellates	*Florenciella*			X				X				
Phytoflagellates	*Goniomonas*			X				X				
Phytoflagellates	*Halosphaera*					X						
Phytoflagellates	*Haptolina*		X	X			X	X			X	
Phytoflagellates	*Helgoeca*		X				X				X	
Phytoflagellates	*Hemiselmis*		X	X			X	X			X	
Phytoflagellates	*Hemistasia*		X				X					
Phytoflagellates	*Hermesinum*	X										
Phytoflagellates	*Hicanonectes*			X								
Phytoflagellates	*Kalinella*						X					
Phytoflagellates	*Kathablepharis*			X								
Phytoflagellates	*Lepidochromonas*							X				
Phytoflagellates	*Leucocryptos*	X		X				X			X	
Phytoflagellates	*Lotharella*		X	X			X	X			X	
Phytoflagellates	*Mamiella*		X	X			X	X			X	
Phytoflagellates	*Mantoniella*		X	X			X	X				
Phytoflagellates	*Marsupiomonas*							X				
Phytoflagellates	*Melkoniania*						X	X			X	
Phytoflagellates	*Meringosphaera*	X								X		
Phytoflagellates	*Micromonas*		X	X			X	X			X	
Phytoflagellates	*Microrhizoidea*						X					
Phytoflagellates	*Minorisa*		X	X			X	X			X	

Phytoflagellates	*Monoraphidium*							X				
Phytoflagellates	*Monorhizochytrium*			X								
Phytoflagellates	*Mychonastes*						X	X				
Phytoflagellates	*Nannochloropsis*						X	X				
Phytoflagellates	*Neobodo*		X									
Phytoflagellates	*Nephroselmis*		X	X			X				X	
Phytoflagellates	*Norrisiella*			X								
Phytoflagellates	*Notosolenus*		X									
Phytoflagellates	*Oblongichytrium*		X	X			X	X				
Phytoflagellates	*Octactis*	X	X	X			X	X		X	X	
Phytoflagellates	*Ollicola*	X										
Phytoflagellates	*Ostreococcus*		X	X			X	X			X	
Phytoflagellates	*Pachysphaera*	X										
Phytoflagellates	*Parabodo*		X									
Phytoflagellates	*Paraphysomonas*		X	X			X	X			X	
Phytoflagellates	*Partenskyella*		X				X				X	
Phytoflagellates	*Paulinella*	X	X	X			X	X			X	
Phytoflagellates	*Pavlomulina*		X	X								
Phytoflagellates	*Pavlova*		X	X								
Phytoflagellates	*Pelagomonas*		X	X			X	X			X	
Phytoflagellates	*Percolomonas*		X				X					
Phytoflagellates	*Phaeocystis*		X	X			X	X			X	
Phytoflagellates	*Phaeomonas*						X	X				
Phytoflagellates	*Picochlorum*		X	X			X	X				
Phytoflagellates	*Pinguiochrysis*		X	X							X	
Phytoflagellates	*Pirsonia*			X								
Phytoflagellates	*Plagioselmis*		X	X			X	X			X	
Phytoflagellates	*Pleurasiga*							X				
Phytoflagellates	*Poterioochromonas*						X	X				
Phytoflagellates	*Poteriospumella*						X					
Phytoflagellates	*Prasinoderma*		X	X								
Phytoflagellates	*Prasinopapilla*							X				
Phytoflagellates	*Procryptobia*		X									
Phytoflagellates	*Protaspa*		X				X				X	
Phytoflagellates	*Proteomonas*		X	X			X	X			X	
Phytoflagellates	*Prototheca*						X					
Phytoflagellates	*Prymnesium*		X	X			X	X			X	
Phytoflagellates	*Pseudobodo*		X	X			X				X	
Phytoflagellates	*Pseudochattonella*		X	X			X	X			X	
Phytoflagellates	*Pseudopedinella*			X			X	X			X	
Phytoflagellates	*Pseudoscourfieldia*	X	X	X			X	X			X	
Phytoflagellates	*Pseudostephanoeca*			X								
Phytoflagellates	*Pteridomonas*		X								X	
Phytoflagellates	*Pterosperma*			X				X				
Phytoflagellates	*Pyramimonas*	X	X	X			X	X			X	
Phytoflagellates	*Quadricilia*			X								
Phytoflagellates	*Rapaza*		X				X					

Phytoflagellates	*Rhinomonas*		X								X	
Phytoflagellates	*Rhizochromulina*		X				X				X	
Phytoflagellates	*Rhodelphis*			X				X				
Phytoflagellates	*Rhodomonas*			X				X				
Phytoflagellates	*Rhogostoma*		X									
Phytoflagellates	*Rhynchomonas*						X					
Phytoflagellates	*Sandona*			X								
Phytoflagellates	*Sarcinochrysis*			X				X				
Phytoflagellates	*Savillea*							X				
Phytoflagellates	*Sorodiplophrys*		X				X					
Phytoflagellates	*Spumella*		X	X			X					
Phytoflagellates	*Teleaulax*		X	X			X	X			X	
Phytoflagellates	*Telonema*		X	X			X	X			X	
Phytoflagellates	*Tetracystis*		X									
Phytoflagellates	*Tetraselmis*	X		X			X	X			X	
Phytoflagellates	*Thalassomyxa*			X				X				
Phytoflagellates	*Thraustochytrium*		X									
Phytoflagellates	*Trebouxia*		X	X			X					
Phytoflagellates	*Triparma*		X	X	X		X	X			X	X
Phytoflagellates	*Ventrifissura*		X	X			X	X			X	

Table S6. List of species recorded in the three sites combining LM and MB.

		C1				SG1				00BF		
**Group**	**Species**	**LM**	**V9**	**V4**	***rbcL***	**LM**	**V9**	**V4**	***rbcL***	**LM**	**V9**	***rbcL***
Coccolithophores	*Acanthoica quattrospina*	X										
Coccolithophores	*Algirosphaera robusta*			X				X				
Coccolithophores	*Braarudosphaera bigelowii*		X				X				X	
Coccolithophores	*Calciosolenia brasiliensis*	X				X				X		
Coccolithophores	*Calciosolenia murrayi*	X				X				X		
Coccolithophores	*Calyptrosphaera oblonga*					X				X		
Coccolithophores	*Emiliania huxleyi*	X	X			X	X			X	X	
Coccolithophores	*Michaelsarsia adriatica*					X						
Coccolithophores	*Michaelsarsia elegans*					X						
Coccolithophores	*Ophiaster hydroideus*	X								X		
Coccolithophores	*Rhabdosphaera clavigera*	X				X				X		
Coccolithophores	*Syracosphaera histrica*									X		
Coccolithophores	*Syracosphaera mediterranea*	X		X								
Coccolithophores	*Syracosphaera pulchra*	X		X		X		X		X		
Diatoms	*Achnanthidium anastasiae*				X							
Diatoms	*Achnanthidium minutissimum*				X				X			
Diatoms	*Adlafia minuscula*								X			
Diatoms	*Amphora grevilleana*				X							
Diatoms	*Amphora helenensis*				X							

Diatoms	*Amphora ovalis*							X	X			
Diatoms	*Amphora sublaevis*				X							
Diatoms	*Arcocellulus mammifer*								X			
Diatoms	*Asterionellopsis glacialis*	X			X							
Diatoms	*Asterionellopsis guyunusae*				X				X			
Diatoms	*Asteromphalus hookeri*					X						
Diatoms	*Bacillaria paxillifer*				X							
Diatoms	*Bacteriastrum delicatulum*									X		
Diatoms	*Bacteriastrum furcatum*	X										
Diatoms	*Bacteriastrum hyalinum*			X								
Diatoms	*Bacteriastrum jadranum*	X										
Diatoms	*Bacteriastrum mediterraneum*	X	X									
Diatoms	*Berkeleya fennica*				X				X			
Diatoms	*Berkeleya hyalina*				X							
Diatoms	*Ceratanaulus creticus*						X					
Diatoms	*Cerataulina pelagica*	X		X	X	X		X	X	X		X
Diatoms	*Chaetoceros affinis*	X	X			X	X	X		X	X	
Diatoms	*Chaetoceros anastomosans*	X			X			X	X			
Diatoms	*Chaetoceros brevis*	X	X	X	X		X	X	X			
Diatoms	*Chaetoceros calcitrans*				X				X			
Diatoms	*Chaetoceros compressus*					X						
Diatoms	*Chaetoceros constrictus*				X				X			X
Diatoms	*Chaetoceros costatus*	X		X	X			X	X			
Diatoms	*Chaetoceros curvisetus*	X		X	X	X	X	X	X	X		
Diatoms	*Chaetoceros dadayi*	X										
Diatoms	*Chaetoceros danicus*	X		X	X	X		X	X			X
Diatoms	*Chaetoceros decipiens*			X	X			X	X			X
Diatoms	*Chaetoceros diadema*				X				X			
Diatoms	*Chaetoceros didymus*	X				X						
Diatoms	*Chaetoceros diversus*				X	X		X	X			
Diatoms	*Chaetoceros eibenii*											X
Diatoms	*Chaetoceros lauderi*		X	X	X		X	X				X
Diatoms	*Chaetoceros lorenzianus*	X				X						
Diatoms	*Chaetoceros minimus*							X				
Diatoms	*Chaetoceros muelleri*		X	X			X	X				
Diatoms	*Chaetoceros protuberans*			X			X	X				
Diatoms	*Chaetoceros pumilum*			X								
Diatoms	*Chaetoceros rostratus*	X	X		X				X			
Diatoms	*Chaetoceros simplex*		X							X		
Diatoms	*Chaetoceros socialis*	X	X		X		X		X			X
Diatoms	*Chaetoceros tenuissimus*	X	X		X	X	X		X			X
Diatoms	*Chaetoceros teres*	X										
Diatoms	*Chaetoceros tetrastichon*					X						
Diatoms	*Chaetoceros throndsenii*	X	X	X	X	X	X	X	X		X	X
Diatoms	*Chaetoceros tortissimus*	X			X	X			X			
Diatoms	*Chaetoceros vixvisibilis*			X	X				X			X
Diatoms	*Climaconeis lorenzii*											X

Diatoms	*Climaconeis riddleae*				X							
Diatoms	*Cocconeis cupulifera*				X							
Diatoms	*Cocconeis mascarenica*				X							
Diatoms	*Conticribra guillardii*		X		X							
Diatoms	*Coscinodiscus concinnus*											X
Diatoms	*Coscinodiscus radiatus*		X	X	X				X		X	X
Diatoms	*Coscinodiscus wailesii*		X									
Diatoms	*Craspedostauros alyoubii*				X							
Diatoms	*Craticula subminuscula*								X			
Diatoms	*Cyclotella atomus*				X							
Diatoms	*Cyclotella choctawhatcheeana*		X				X				X	
Diatoms	*Cyclotella litoralis*			X								
Diatoms	*Cyclotella striata*		X		X		X		X			
Diatoms	*Cylindrotheca closterium*	X	X	X	X	X	X		X	X		X
Diatoms	*Cymbella cymbiformis*								X			
Diatoms	*Cymbella neocistula*								X			
Diatoms	*Dactyliosolen blavyanus*	X				X						
Diatoms	*Dactyliosolen fragilissimus*	X				X				X		
Diatoms	*Dactyliosolen phuketensis*	X										
Diatoms	*Diatoma moniliformis*								X			
Diatoms	*Diploneis crabro*									X		
Diatoms	*Ditylum brightwellii*	X					X		X			
Diatoms	*Ditylum intricatum*				X							X
Diatoms	*Encyonema silesiacum*								X			
Diatoms	*Encyonema ventricosum*								X			
Diatoms	*Entomoneis paludosa*				X							
Diatoms	*Eolimna minima*				X							
Diatoms	*Epithemia iriomotensis*				X							
Diatoms	*Eucampia cornuta*				X				X			
Diatoms	*Eucampia zodiacus*	X										
Diatoms	*Extubocellulus cribriger*				X							
Diatoms	*Extubocellulus spinifer*				X							
Diatoms	*Fallacia forcipata*				X							
Diatoms	*Fistulifera saprophila*							X	X			
Diatoms	*Gomphonema micropus*								X			
Diatoms	*Gomphonema pumilum*								X			
Diatoms	*Gomphonema saprophilum*								X			
Diatoms	*Gomphonema tergestinum*								X			
Diatoms	*Guinardia delicatula*			X				X				
Diatoms	*Guinardia flaccida*	X		X						X		
Diatoms	*Guinardia striata*	X			X	X			X	X		X
Diatoms	*Gyrosigma acuminatum*				X				X			
Diatoms	*Haslea nipkowii*											X
Diatoms	*Haslea ostrearia*				X							X
Diatoms	*Haslea pseudostrearia*				X							
Diatoms	*Haslea wawrikae*	X										
Diatoms	*Hemiaulus chinensis*	X			X				X			X

Diatoms	*Hemiaulus hauckii*	X								X		
Diatoms	*Homoeocladia volvendirostrata*				X							
Diatoms	*Lauderia annulata*		X		X		X		X		X	
Diatoms	*Leptocylindrus aporus*		X	X			X	X			X	
Diatoms	*Leptocylindrus convexus*		X	X		X						
Diatoms	*Leptocylindrus danicus*		X				X			X	X	
Diatoms	*Leptocylindrus mediterraneus*	X								X		
Diatoms	*Licmophora abbreviata*				X				X			X
Diatoms	*Licmophora colosalis*				X							
Diatoms	*Licmophora paradoxa*				X							
Diatoms	*Licmophora remulus*				X							
Diatoms	*Lithodesmium undulatum*		X									
Diatoms	*Mayamaea permitis*				X		X		X			
Diatoms	*Mediolabrus comicus*			X				X				
Diatoms	*Melosira varians*						X		X			
Diatoms	*Meridion circulare*				X							
Diatoms	*Minidiscus trioculatus*				X				X			X
Diatoms	*Minidiscus variabilis*		X	X								
Diatoms	*Minutocellus polymorphus*				X				X			X
Diatoms	*Navicula perminuta*				X				X			
Diatoms	*Navicula phyllepta*						X	X	X			
Diatoms	*Navicula ramosissima*				X							
Diatoms	*Navicula tripunctata*				X							
Diatoms	*Navicula tsukamotoi*											X
Diatoms	*Nitzschia aequorea*				X							X
Diatoms	*Nitzschia aurariae*								X			
Diatoms	*Nitzschia bulnheimiana*				X							
Diatoms	*Nitzschia fonticola*				X							
Diatoms	*Nitzschia frustulum*				X							X
Diatoms	*Nitzschia gobbii*					X						
Diatoms	*Nitzschia inconspicua*				X			X	X			
Diatoms	*Nitzschia longissima*			X		X				X		
Diatoms	*Nitzschia microcephala*				X		X	X	X			
Diatoms	*Nitzschia palea*								X			
Diatoms	*Nitzschia perminuta*											X
Diatoms	*Nitzschia pusilla*				X				X			
Diatoms	*Nitzschia sigma*				X	X						
Diatoms	*Nitzschia sigmoidea*				X							
Diatoms	*Nitzschia supralitorea*				X				X			
Diatoms	*Nitzschia valdestriata*				X							
Diatoms	*Papiliocellulus elegans*			X				X			X	
Diatoms	*Papiliocellulus simplex*				X				X			X
Diatoms	*Paralia sulcata*				X				X			X
Diatoms	*Pleurosigma intermedium*		X				X				X	
Diatoms	*Pleurosigma normanii*									X		
Diatoms	*Proboscia alata*	X				X				X		
Diatoms	*Proboscia indica*	X								X		

Diatoms	*Psammodictyon constrictum*				X							X
Diatoms	*Pseudo-nitzschia australis*						X					
Diatoms	*Pseudo-nitzschia calliantha*				X				X	X		
Diatoms	*Pseudo-nitzschia delicatissima*				X	X			X			X
Diatoms	*Pseudo-nitzschia fraudulenta*								X	X		
Diatoms	*Pseudo-nitzschia galaxiae*				X				X			X
Diatoms	*Pseudo-nitzschia heimii*										X	
Diatoms	*Pseudo-nitzschia multiseries*				X							
Diatoms	*Pseudo-nitzschia pseudodelicatissima*					X						
Diatoms	*Pseudo-nitzschia pungens*				X			X	X			
Diatoms	*Pseudo-nitzschia subfraudulenta*				X							X
Diatoms	*Pseudosolenia calcar-avis*	X			X			X	X			
Diatoms	*Pseudostaurosira elliptica*				X							
Diatoms	*Ralfsiella smithii*								X			
Diatoms	*Rhizosolenia fallax*				X				X			X
Diatoms	*Rhizosolenia imbricata*				X			X	X			X
Diatoms	*Sellaphora seminulum*				X							
Diatoms	*Seminavis robusta*				X							
Diatoms	*Shionodiscus oestrupii*				X							
Diatoms	*Skeletonema costatum*				X				X	X		
Diatoms	*Skeletonema marinoi*		X	X		X	X	X			X	
Diatoms	*Skeletonema menzelii*	X					X				X	X
Diatoms	*Skeletonema pseudocostatum*		X									
Diatoms	*Staurosirella leptostauron*				X							
Diatoms	*Stephanocyclus gamma*				X				X			X
Diatoms	*Stephanocyclus meneghinianus*								X			
Diatoms	*Striatella unipunctata*				X				X			
Diatoms	*Surirella librile*								X			
Diatoms	*Surirella minuta*								X			
Diatoms	*Tabularia laevis*				X							
Diatoms	*Talaroneis posidoniae*				X							X
Diatoms	*Tenuicylindrus belgicus*						X					
Diatoms	*Thalassionema frauenfeldii*				X				X			X
Diatoms	*Thalassionema nitzschioides*					X				X		
Diatoms	*Thalassiosira eccentrica*				X							X
Diatoms	*Thalassiosira gravida*				X		X		X			
Diatoms	*Thalassiosira mediterranea*				X				X			X
Diatoms	*Thalassiosira minima*				X				X			
Diatoms	*Thalassiosira nordenskioeldii*				X							
Diatoms	*Thalassiosira oceanica*				X				X			X
Diatoms	*Thalassiosira profunda*			X	X			X	X			X
Diatoms	*Thalassiosira pseudonana*		X	X	X				X			
Diatoms	*Thalassiosira punctigera*				X				X			
Diatoms	*Thalassiosira rotula*	X				X						
Diatoms	*Toxarium hennedyanum*				X							X
Diatoms	*Tryblionella apiculata*				X							
Diatoms	*Ulnaria ulna*								X			

Dinoflagellates	*Akashiwo sanguinea*					X	X	X				
Dinoflagellates	*Alexandrium affine*		X	X								
Dinoflagellates	*Alexandrium andersonii*		X	X			X				X	
Dinoflagellates	*Alexandrium leei*		X	X								
Dinoflagellates	*Alexandrium margalefii*		X	X			X	X			X	
Dinoflagellates	*Alexandrium minutum*		X				X	X		X	X	
Dinoflagellates	*Alexandrium pseudogonyaulax*	X	X				X			X	X	
Dinoflagellates	*Amoebophrya ceratii*		X				X				X	
Dinoflagellates	*Amylax triacantha*			X								
Dinoflagellates	*Ansanella granifera*		X				X				X	
Dinoflagellates	*Ataxiodinium choane*			X								
Dinoflagellates	*Azadinium spinosum*							X				
Dinoflagellates	*Balechina gracilis*			X								
Dinoflagellates	*Biecheleria brevisulcata*						X					
Dinoflagellates	*Biecheleria cincta*		X	X			X	X			X	
Dinoflagellates	*Biecheleriopsis adriatica*		X	X			X	X			X	
Dinoflagellates	*Bispinodinium angelaceum*										X	
Dinoflagellates	*Blastodinium contortum*		X	X								
Dinoflagellates	*Blastodinium galatheanum*		X								X	
Dinoflagellates	*Blastodinium mangini*		X	X			X				X	
Dinoflagellates	*Blastodinium spinulosum*		X					X				
Dinoflagellates	*Blixaea quinquecornis*							X				
Dinoflagellates	*Ceratoperidinium falcatum*	X										
Dinoflagellates	*Ceratoperidinium margalefii*	X										
Dinoflagellates	*Chytriodinium affine*			X								
Dinoflagellates	*Cucumeridinium coeruleum*			X								
Dinoflagellates	*Dinophysis acuminata*		X									
Dinoflagellates	*Dinophysis caudata*	X								X		
Dinoflagellates	*Dinophysis fortii*	X								X		
Dinoflagellates	*Dinophysis sacculus*					X						
Dinoflagellates	*Durinskia dybowskii*		X	X								
Dinoflagellates	*Ellobiopsis chattonii*		X	X				X			X	
Dinoflagellates	*Euduboscquella costata*			X				X				
Dinoflagellates	*Fragilidium duplocampaniforme*		X	X			X	X			X	
Dinoflagellates	*Fragilidium mexicanum*		X	X			X	X			X	
Dinoflagellates	*Gonyaulax bohaiensis*			X				X				
Dinoflagellates	*Gonyaulax cochlea*			X								
Dinoflagellates	*Gonyaulax digitalis*		X	X			X	X			X	
Dinoflagellates	*Gonyaulax ellegaardiae*			X								
Dinoflagellates	*Gonyaulax fragilis*			X				X				
Dinoflagellates	*Gonyaulax polygramma*		X	X			X	X			X	
Dinoflagellates	*Gonyaulax portimonensis*			X								
Dinoflagellates	*Gonyaulax spinifera*	X	X	X		X	X				X	
Dinoflagellates	*Gonyaulax whaseongensis*		X				X	X			X	
Dinoflagellates	*Grammatodinium tongyeonginum*							X				
Dinoflagellates	*Gymnodinium catenatum*		X				X	X				
Dinoflagellates	*Gymnodinium dorsalisulcum*			X				X				

Dinoflagellates	*Gymnodinium impudicum*						X	X				
Dinoflagellates	*Gymnoxanthella radiolariae*		X				X					
Dinoflagellates	*Gyrodiniellum shiwhaense*		X				X				X	
Dinoflagellates	*Gyrodinium dominans*			X				X				
Dinoflagellates	*Gyrodinium fusiforme*							X				
Dinoflagellates	*Gyrodinium heterogrammum*			X								
Dinoflagellates	*Gyrodinium jinhaense*			X				X				
Dinoflagellates	*Heterocapsa nei/rotundata*		X	X			X	X			X	
Dinoflagellates	*Heterocapsa pygmaea*		X				X				X	
Dinoflagellates	*Islandinium tricingulatum*		X	X			X	X			X	
Dinoflagellates	*Karenia mikimotoi*		X				X					
Dinoflagellates	*Karenia papilionacea*					X						
Dinoflagellates	*Karlodinium veneficum*		X				X					
Dinoflagellates	*Kofoidinium pavillardii*			X				X				
Dinoflagellates	*Kofoidinium velleloides*	X				X						
Dinoflagellates	*Lepidodinium viride*		X				X				X	
Dinoflagellates	*Lessardia elongata*	X						X				
Dinoflagellates	*Levanderina fissa*						X	X				
Dinoflagellates	*Lingulaulax polyedra*		X	X		X	X				X	
Dinoflagellates	*Margalefidinium fulvescens*		X	X			X	X			X	
Dinoflagellates	*Noctiluca scintillans*	X	X	X		X	X	X			X	
Dinoflagellates	*Nusuttodinium desymbiontum*		X									
Dinoflagellates	*Ostreopsis ovata*		X	X								
Dinoflagellates	*Oxyphysis oxytoxoides*					X						
Dinoflagellates	*Oxytoxum caudatum*	X				X						
Dinoflagellates	*Oxytoxum elongatum*					X						
Dinoflagellates	*Oxytoxum laticeps*					X						
Dinoflagellates	*Oxytoxum milneri*	X										
Dinoflagellates	*Oxytoxum scolopax*					X						
Dinoflagellates	*Oxytoxum variabile*	X										
Dinoflagellates	*Paragymnodinium shiwhaense*		X				X				X	
Dinoflagellates	*Paulsenella vonstoschii*		X				X				X	
Dinoflagellates	*Pelagodinium bei*		X	X			X	X			X	
Dinoflagellates	*Pentapharsodinium tyrrhenicum*										X	
Dinoflagellates	*Phalacroma doryphorum*			X								
Dinoflagellates	*Phalacroma oxytoxoides*		X				X					
Dinoflagellates	*Phalacroma rotundatum*					X						
Dinoflagellates	*Podolampas palmipes*	X										
Dinoflagellates	*Polykrikos geminatus*		X				X	X			X	
Dinoflagellates	*Polykrikos hartmannii*		X				X					
Dinoflagellates	*Polykrikos kofoidii*			X				X				
Dinoflagellates	*Polykrikos schwartzii*							X				
Dinoflagellates	*Posoniella tricarinelloides*		X									
Dinoflagellates	*Prorocentrum balticum*									X		
Dinoflagellates	*Prorocentrum bidens*	X				X				X		
Dinoflagellates	*Prorocentrum cordatum*	X	X			X	X					
Dinoflagellates	*Prorocentrum dactylus*	X										

Dinoflagellates	*Prorocentrum gracile*									X		
Dinoflagellates	*Prorocentrum micans*	X				X				X		
Dinoflagellates	*Prorocentrum minimum*									X		
Dinoflagellates	*Prorocentrum triestinum*					X				X		
Dinoflagellates	*Prorocentrum vaginulum*	X										
Dinoflagellates	*Protoceratium reticulatum*	X	X	X		X	X	X				
Dinoflagellates	*Protodinium simplex*							X				
Dinoflagellates	*Protoperidinium bipes*	X		X		X		X		X		
Dinoflagellates	*Protoperidinium claudicans*					X						
Dinoflagellates	*Protoperidinium depressum*			X								
Dinoflagellates	*Protoperidinium diabolus*	X				X						
Dinoflagellates	*Protoperidinium divergens*					X						
Dinoflagellates	*Protoperidinium monovelum*			X								
Dinoflagellates	*Protoperidinium ovum*					X						
Dinoflagellates	*Protoperidinium steinii*					X						
Dinoflagellates	*Pseliodinium fusus*			X				X				
Dinoflagellates	*Pyrocystis lunula*		X	X								
Dinoflagellates	*Pyrophacus steinii*							X				
Dinoflagellates	*Qia lebouriae*			X								
Dinoflagellates	*Schuettiella mitra*		X	X							X	
Dinoflagellates	*Scrippsiella acuminata*			X				X				
Dinoflagellates	*Scrippsiella trochoidea*									X		
Dinoflagellates	*Spatulodinium pseudonoctiluca*			X				X				
Dinoflagellates	*Stoeckeria algicida*		X	X								
Dinoflagellates	*Syltodinium listii*			X				X				
Dinoflagellates	*Torodinium robustum*	X										
Dinoflagellates	*Triadinium polyedricum*	X	X	X			X				X	
Dinoflagellates	*Tripos candelabrum*									X		
Dinoflagellates	*Tripos extensus*	X				X						
Dinoflagellates	*Tripos furca*	X	X	X		X	X	X			X	
Dinoflagellates	*Tripos fusus*	X	X	X		X	X	X		X	X	
Dinoflagellates	*Tripos hexacanthus*			X								
Dinoflagellates	*Tripos kofoidii*	X										
Dinoflagellates	*Tripos macroceros*	X										
Dinoflagellates	*Tripos massiliensis*			X								
Dinoflagellates	*Tripos tenuis*		X				X				X	
Dinoflagellates	*Tripos trichoceros*	X										
Dinoflagellates	*Wangodinium sinense*			X				X				
Dinoflagellates	*Yihiella yeosuensis*		X	X			X	X			X	
Dinoflagellates	*Zooxanthella nutricula*		X									
Phytoflagellates	*Allobodo chlorophagus*		X									
Phytoflagellates	*Allovahlkampfia spelaea*						X					
Phytoflagellates	*Apedinella radians*	X		X			X	X				
Phytoflagellates	*Aplanochytrium minutum*			X								
Phytoflagellates	*Apusomonas proboscidea*							X				
Phytoflagellates	*Aureococcus anophagefferens*		X	X			X	X			X	
Phytoflagellates	*Baffinella frigidus*		X									

Phytoflagellates	*Bathycoccus prasinos*		X	X			X	X			X	
Phytoflagellates	*Bicosoeca vacillans*		X	X			X	X			X	
Phytoflagellates	*Bicosta minor*			X				X				
Phytoflagellates	*Bigelowiella natans*						X				X	
Phytoflagellates	*Botuliforma benthica*		X				X					
Phytoflagellates	*Cafeteria baltica*										X	
Phytoflagellates	*Calliacantha natans*			X								
Phytoflagellates	*Chattonella marina*						X					
Phytoflagellates	*Chattonella subsalsa*							X				
Phytoflagellates	*Chlamydomonas acidophila*		X									
Phytoflagellates	*Chlamydomonas hedleyi*		X									
Phytoflagellates	*Chlamydomonas kuwadae*			X				X				
Phytoflagellates	*Chlamydomonas parkeae*			X								
Phytoflagellates	*Chlorarachnion reptans*		X	X			X	X				
Phytoflagellates	*Chlorochytrium lemnae*						X					
Phytoflagellates	*Chloroparvula pacifica*		X	X			X	X			X	
Phytoflagellates	*Chrysochromulina campanulifera*		X				X				X	
Phytoflagellates	*Chrysochromulina cymbium*						X				X	
Phytoflagellates	*Chrysochromulina leadbeateri*		X	X			X	X			X	
Phytoflagellates	*Chrysochromulina parva*		X				X					
Phytoflagellates	*Chrysochromulina rotalis*		X	X			X	X				
Phytoflagellates	*Chrysochromulina scutellum*		X				X				X	
Phytoflagellates	*Chrysochromulina simplex*		X				X				X	
Phytoflagellates	*Chrysochromulina spinifera*		X				X	X			X	
Phytoflagellates	*Chrysolepidomonas dendrolepidota*			X				X				
Phytoflagellates	*Ciliophrys infusionum*		X								X	
Phytoflagellates	*Commation cryoporinum*	X										
Phytoflagellates	*Cryothecomonas aestivalis*		X	X			X	X			X	
Phytoflagellates	*Cymbomonas tetramitiformis*		X	X			X	X				
Phytoflagellates	*Desmodesmus abundans*						X					
Phytoflagellates	*Desmodesmus bicellularis*						X					
Phytoflagellates	*Desmodesmus subspicatus*						X					
Phytoflagellates	*Dicrateria rotunda*			X								
Phytoflagellates	*Dictyocha fibula*	X				X						
Phytoflagellates	*Dinobryon coalescens*	X										
Phytoflagellates	*Dinobryon faculiferum*					X						
Phytoflagellates	*Dinobryon porrectum*					X						
Phytoflagellates	*Dinobryon sociale*				X				X			X
Phytoflagellates	*Discomonas retusa*			X								
Phytoflagellates	*Dolichomastix tenuilepis*			X				X				
Phytoflagellates	*Ebria tripartita*		X				X				X	
Phytoflagellates	*Eutreptiella gymnastica*		X									
Phytoflagellates	*Fibrocapsa japonica*						X	X				
Phytoflagellates	*Fibrophrys columna*			X								
Phytoflagellates	*Goniomonas amphinema*							X				
Phytoflagellates	*Haptolina herdlensis*		X				X				X	
Phytoflagellates	*Helgoeca nana*		X				X				X	

Phytoflagellates	*Hemiselmis aquamarina*		X				X	X			X	
Phytoflagellates	*Hemiselmis cryptochromatica*			X								
Phytoflagellates	*Hemiselmis virescens*						X					
Phytoflagellates	*Hermesinum adriaticum*	X										
Phytoflagellates	*Hicanonectes teleskopos*			X								
Phytoflagellates	*Kathablepharis japonica*			X								
Phytoflagellates	*Leucocryptos marina*	X		X				X			X	
Phytoflagellates	*Lotharella vacuolata*		X				X				X	
Phytoflagellates	*Mamiella gilva*		X	X			X	X			X	
Phytoflagellates	*Mantoniella squamata*		X	X			X	X				
Phytoflagellates	*Meringosphaera mediterranea*	X								X		
Phytoflagellates	*Micromonas bravo*		X	X			X	X			X	
Phytoflagellates	*Micromonas commoda*		X	X			X	X				
Phytoflagellates	*Microrhizoidea pickettheapsiorum*						X					
Phytoflagellates	*Minorisa minuta*		X	X			X	X			X	
Phytoflagellates	*Nematochrysis hieroglyphica*			X								
Phytoflagellates	*Nephroselmis astigmatica*						X				X	
Phytoflagellates	*Nephroselmis pyriformis*			X								
Phytoflagellates	*Nephroselmis rotunda*		X								X	
Phytoflagellates	*Norisiella sphaerica*			X								
Phytoflagellates	*Notosolenus urceolatus*		X									
Phytoflagellates	*Octactis octonaria*	X										
Phytoflagellates	*Octactis speculum*		X	X			X	X		X	X	
Phytoflagellates	*Ollicola vangoorii*	X										
Phytoflagellates	*Ostreococcus mediterraneus*			X				X				
Phytoflagellates	*Ostreococcus tauri*			X				X				
Phytoflagellates	*Paraphysomonas foraminifera*		X								X	
Phytoflagellates	*Partenskyella glossopodia*		X				X				X	
Phytoflagellates	*Paulinella chromatophora*						X					
Phytoflagellates	*Paulinella ovalis*	X										
Phytoflagellates	*Pavlomulina ranunculiformis*		X									
Phytoflagellates	*Pavlova pinguis*			X								
Phytoflagellates	*Pedinella elastica*			X			X	X			X	
Phytoflagellates	*Pedospumella elongata*			X								
Phytoflagellates	*Pelagomonas calceolata*		X	X			X	X			X	
Phytoflagellates	*Percolomonas cosmopolita*						X					
Phytoflagellates	*Phaeocystis cordata*		X	X			X	X			X	
Phytoflagellates	*Phaeocystis globosa*			X								
Phytoflagellates	*Phaeocystis jahnii*			X								
Phytoflagellates	*Phaeomonas parva*						X	X				
Phytoflagellates	*Picochlorum maculatum*						X					
Phytoflagellates	*Pinguiochrysis pyriformis*		X	X							X	
Phytoflagellates	*Pirsonia formosa*			X								
Phytoflagellates	*Pleurasiga minima*							X				
Phytoflagellates	*Poterioochromonas malhamensis*							X				
Phytoflagellates	*Prasinoderma coloniale*			X								
Phytoflagellates	*Prasinopapilla vacuolata*							X				

Phytoflagellates	*Protaspa grandis*		X									
Phytoflagellates	*Protaspa obliqua*		X									
Phytoflagellates	*Proteomonas sulcata*		X				X					
Phytoflagellates	*Prymnesium palpebrale*		X				X				X	
Phytoflagellates	*Pseudobodo tremulans*		X				X				X	
Phytoflagellates	*Pseudochattonella verruculosa*		X				X					
Phytoflagellates	*Pseudoscourfieldia marina*	X	X	X			X	X			X	
Phytoflagellates	*Pseudostephanoeca paucicostata*			X								
Phytoflagellates	*Pterosperma cristatum*			X				X				
Phytoflagellates	*Pyramimonas australis*			X								
Phytoflagellates	*Pyramimonas parkeae*		X				X				X	
Phytoflagellates	*Pyramimonas propulsa*			X								
Phytoflagellates	*Pyramimonas tetrarhynchus*		X				X				X	
Phytoflagellates	*Quadricilia rotundata*			X								
Phytoflagellates	*Rapaza viridis*		X				X					
Phytoflagellates	*Rhizochromulina marina*		X				X				X	
Phytoflagellates	*Rhodelphis marina*			X				X				
Phytoflagellates	*Rhynchomonas nasuta*						X					
Phytoflagellates	*Savillea parva*							X				
Phytoflagellates	*Sorodiplophrys stercorea*		X				X					
Phytoflagellates	*Spumella lacusvadosi*						X					
Phytoflagellates	*Spumella vulgaris*		X				X					
Phytoflagellates	*Teleaulax acuta*		X	X			X	X			X	
Phytoflagellates	*Teleaulax amphioxeia*		X	X			X	X			X	
Phytoflagellates	*Telonema antarcticum*		X	X			X	X			X	
Phytoflagellates	*Telonema subtile*		X	X			X	X			X	
Phytoflagellates	*Tetracystis pampae*		X									
Phytoflagellates	*Tetraselmis convolutae*			X				X				
Phytoflagellates	*Thraustochytrium kinnei*		X									
Phytoflagellates	*Trebouxia crenulata*						X					
Phytoflagellates	*Trebouxia impressa*						X					
Phytoflagellates	*Triparma eleuthera*						X	X				
Phytoflagellates	*Triparma laevis*			X				X				
Phytoflagellates	*Triparma mediterranea*		X	X			X	X			X	
Phytoflagellates	*Triparma pacifica*		X	X	X		X	X			X	X
Phytoflagellates	*Ventrifissura artocarpoidea*		X				X	X				
Phytoflagellates	*Ventrifissura foliiformis*		X				X				X	
Phytoflagellates	*Ventrifissura oblonga*			X								
Phytoflagellates	*Vicicitus globosus*			X				X				
